# The Spectrum of Venetoclax-Based Treatments in Acute Myeloid Leukemia

**DOI:** 10.3390/cancers18081201

**Published:** 2026-04-09

**Authors:** Elvira Pelosi, Germana Castelli, Ugo Testa

**Affiliations:** Department of Oncology, Istituto Superiore di Sanità, Viale Regina Elena 299, 00161 Rome, Italy; elvira.pelosi56@gmail.com (E.P.); germana.castelli@iss.it (G.C.)

**Keywords:** venetoclax, acute myeloid leukemia, hypomethylating agents, targeted therapy, genomic profiling, clinical studies

## Abstract

**Background/Objectives**: In recent years there has been a consistent development of clinical studies surrounding the incorporation of the B-cell lymphoma 2 (BCL-2) inhibitor venetoclax (VEN) into the treatment of acute myeloid leukemia (AML) **Methods**: A search of the literature showed a tremendous development of experimental and clinical studies evaluating the impact of VEN-based regimens in the treatment of AML patients. This review comprehensively analyzes the available scientific evidence—including prospective clinical trials, retrospective cohorts, and real-world studies—to summarize current knowledge on the efficacy and safety of venetoclax-based regimens in AML patients. **Results**: Recent studies have evaluated VEN-based regimens in newly diagnosed (ND) and refractory/relapsed (R/R) AML patients, showing the efficacy of these treatments. VEN with hypomethylating agents (HMAs) became the standard-of-care for elderly/unfit AML patients. Recent studies strongly support the effectiveness of VEN-based regimens in frontline treatment of adult AML patients eligible for intensive treatments. VEN-based therapies were also used in combination with targeted therapies, thus generating triplet therapeutic regimens that are under evaluation for the treatment of some AML subtypes. However, the response to VEN+HMAs is highly variable and in part depends on tumor genetics; some patients are resistant or relapse following VEN-based treatments and future studies will be required to develop therapeutic strategies able to circumvent resistance and to identify patients at high risk of relapse. Prospective randomized trials are required to establish the real efficacy of VEN in various clinical settings and to refine maintenance and discontinuation strategies, aiming to improve long-term outcomes and to make more safe treatments based on VEN.

## Simple Summary

Venetoclax has proven to be an effective therapy for newly diagnosed, relapsed and refractory AML patients unfit for induction chemotherapy. Subsequent clinical trials have expanded the clinical evaluation of venetoclax in various drug combinations and in the treatment of different types of AML patients regarding age, disease stage and fitness for intensive treatments. However, response rates of patients treated with venetoclax regimens vary widely, depending on mutational profile and patient characteristics. This review attempts to provide an overview of the wide spectrum of growing clinical applications of venetoclax-based regimens. Current ongoing clinical trials are evaluating various venetoclax-based drug combinations in different clinical settings in both AML patients eligible and not for intensive treatments. These studies have generated many promising clinical applications of venetoclax-based therapies, but in many instances these observations need to be validated through randomized clinical trials.

## 1. Introduction

Acute myeloid leukemia (AML) is a heterogeneous group of myeloid malignancies characterized by an abnormal proliferation and differentiation of myeloid leukemic blasts in the bone marrow. AML cells display a consistent number of acquired genetic abnormalities allowing the identification of molecular subgroups. AML is typically a disease of older adults, with a median age at diagnosis of >65 years.

Standard treatment for younger patients and fit patients with AML consists of intensive chemotherapy induction, followed by consolidation treatment in patients achieving a remission and allogeneic hematopoietic stem cell transplantation (allo-HSCT) in eligible patients. The treatment of older AML patients is challenging in that only a part of these patients can tolerate high-intensity treatments. The earlier treatment of older AML patients was particularly challenging because these patients have a poor response due to: disease biology (frequent presence of adverse cytogenetics and a higher frequency of secondary and therapy-related AML, less responsive to antileukemic drugs); high treatment-related mortality and toxicity, rendering these patients unfit for intensive chemotherapy, leaving them very limited options with low-dose chemotherapy or hypomethylating agents.

**Figure 1:**
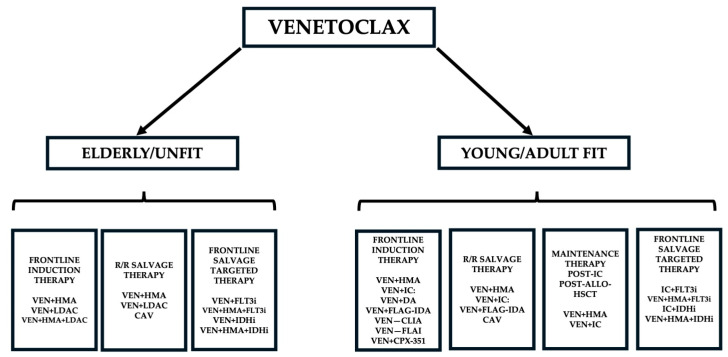
Spectrum of different VEN-based treatments in elderly/unfit and young/adult fit AML patients. The various treatments have been subdivided into therapies for frontline induction or for salvage of R/R AML patients. Abbreviations: IC, intensive chemotherapy; VEN, Venetoclax; HMA, Hypomethylating Agent; FLT3i, FLT3 inhibitor; IDH1, IDH inhibitor; ALLO-HSCT, allogeneic hematopoietic stem cell transplantation; CAV, Cyclophosphamide, Adriamycin, Vincristine; LDAC, Low-dose Ara-C; FALG-IDA, Fludarabine, Leukine (G-CSF), Ara-C, Idarubicin; DA, Daunorubicin and Ara-C; FLAI, Fludarabine, Ara-C, Idarubicin; CLAI, Cladribine, Idarubicin, Ara-C.

The treatment of older AML patients was completely revolutionized by the introduction in therapy of B-cell lymphoma-2 (BCL-2) inhibitors. Venetoclax (VEN) is a BH3-mimetic agent that specifically interacts with the antiapoptotic BCL-2 protein, causing cytochrome c release from mitochondria, consequent caspase activation and apoptosis. Venetoclax (VEN) displayed remarkable antileukemic activity, particularly when administered in association with hypomethylating agents (HMAs), either azacitidine or decitabine. VEN received approval on 21 November 2018 by the Food and Drug Administration for use in older AML patients who are unfit or are age 75 years or older combined with either HMAs or low-dose cytarabine [1,2]. The introduction of VEN in the treatment of older AML patients was a game-changer in AML for older or “unfit” patients with newly diagnosed AML, and the combination of VEN with a HMA has significantly improved survival and, in some patients, serves as a bridge to curative transplant [1,2]. Combinations of lower intensity therapy with VEN have become the standard of care for patients with AML who are age 65 years or older or deemed unfit for intensive therapy [1,2]. The success obtained in these studies has stimulated the study of VEN in combination with other lower-intensity regimens and its introduction into higher-intensity regimens [1,2]. More recent studies have supported the efficacy of VEN-based combinations for the treatment of fit adult AML patients (Figure 1).

The present review provides an updated and detailed analysis of recent studies involving the use of different VEN-based regimens in the treatment of AML patients. Particularly, this review aims to analyze the current direction and research area involving the clinical use of VEN-based therapies, with particular emphasis on: (i) optimizing the use of VEN in patients who have relapsed after frontline treatment; (ii) assessing the safety and the efficacy of the incorporation of VEN into more intensive chemotherapy regimens for younger “fit” patients; (iii) evaluating the efficacy of VEN-based combinations with IDH1-IDH2 and Menin inhibitors to increase the efficacy in subgroups of AML patients; (iv) defining the role of VEN in *NPM1*-mutated AMLs; (v) developing and assessing the safety and the efficacy of combining VEN+HMA with a third agent to target resistance mechanisms, such as *TP53*, *FLT3-ITD*, and *RAS* mutations; (vi) investigating whether patients with sustained remission after NEN-based treatments can safety stop VEN after >12 months of therapy to avoid long-term toxicity.

## 2. Effects of Venetoclax on Newly Diagnosed and Relapsed/Refractory AML Patients Alone or in Association with a Hypomethylating Agent

A phase Ib study (M15-38 study) involved treatment with venetoclax (VEN) and a hypomethylating agent (HMA), either azacitidine (AZA) or decitabine (DEC), in a group of 145 treatment-naïve AML patients with age ≥65 years (median age 74 years) [3]. Sixty-seven percent of all treated patients achieved a composite complete remission rate (CRc defined by complete remission (CR) plus CR with incomplete count recovery) [3]. CRc rates in patients with poor- and intermediate-risk cytogenetics were 60% and 74%, respectively; in *TP53*-mutant AML, CRc rates were 47%, with a median duration of response of 5.6 months; in *FLT3*-mutant AML 72% with a median duration of response of 11 months; in *IDH1*/*IDH2*.mutant AML 71% with a median duration of response not reached; in *NPM1*-mutant AML, 91.5% with a median duration of response not reached [3]. The safety profile was acceptable and included gastrointestinal symptoms, febrile neutropenia, fatigue, hyperkalemia and decreased WBC [3].

In the VIALE-A study, 431 ND AML patients were randomized to receive either VEN+AZA or AZA+Placebo; at a median follow-up of 20.5 months, the median OS was 14.7 months in the VEN+AZA group versus 9.6 months in the control group; the CRc rate was higher in the VEN+AZA group than in the control group (66.4% vs. 28.3%) [2]. The analysis of the rate of CRc in different molecular subgroups, comparing the VEN+AZA arm to the control arm, showed the following results: *IDH1*/*IDH2* mutations 75.4% vs. 10.7%; *FLT3* mutations 72.4% vs. 36.4%; *NPM1* mutations 66.7% vs. 23.5%; *TP53* mutations 55.3% vs. 0% [4]. At the level of OS, the most significant improvement compared to AZA alone was observed for *IDH1*/*IDH2*-mutant patients treated with VEN+AZA [2].

Long-term follow-up analysis of the patients enrolled in the VIALE-A study reported a 2-year, 3-year and 4-year OS of 58%, 29% and 18%, respectively, in the VEN+AZA arm and 41%, 12% and 0% in the AZA+Placebo arm [5]. The most benefit of VEN+AZA over AZA+Placebo was observed for *IDH1*/*IDH2*-mutant patients [5].

Thirty-three patients from VIALE-A and M14-358 trials received allo-HSCT after VEN+HMA therapy [6]. At the time of HSCT, 85% of these patients were in CRc [6]. With a median follow-up of 31.6 months after HSCT, the median OS was 29.9 months and median duration of remission was not reached; the 12-month and 24-month survival rates were 69% and 61%, respectively; among patients in CRc at the time of HSCT, mOS after HSCT was 29.9 months; mOS was not reached among the patients who had MRD-negative response before HSCT and 28.2 months among patients with MRD-positive responses before HSCT [6]. These observations support VEN+HMA as an alternative induction therapy before HSCT for patients with newly diagnosed AML ineligible for IC [6].

A post hoc analysis of VIALE-A and M15-358 trials assessed the outcomes of patients with therapy-related AML (t-AML) or antecedent myelodysplatic syndromes (s-AML) [4]. This analysis showed that: CRc was achieved among patients treated with VEN+HMA in 61% of t-AML, with a duration of response of 25.5 months, compared to 11% with a duration of 8.5 months in the group of patients treated with AZA alone; CR was achieved among patients treated with VEN+HMA in 66% of s-AML with a duration of response of 21.0 months, compared to 31% with a duration of response of 5.8 months in the group treated with HMA alone [7]. The OS was significantly improved in both s-AML and t-AML by VEN+HMA treatment in comparison with HMA alone [7].

A subanalysis of the VIALE-A study showed that patient benefits from VEN+AZA treatment occurred regardless of age of patients or of degree of patient frailty [8].

However, some clinical trial and real-world studies suggest that, for very elderly AML patients with an age >75 years and particularly vulnerable to treatment-related toxicities, a less intensive regimen, such as HMA alone, aiming at controlling disease while minimizing adverse effects, may be more appropriate than remission-targeted therapy, such as VEN+HMA [9,10].

### 2.1. Responses in AML Molecular Subtypes

A pooled subanalysis of the results observed for *FLT3*-mutant patients in the VIALE-A and M15-38 studies showed: a CRc rate for VEN+AZA versus AZA alone for *FLT3*-mutant patients of 67% vs. 36%, with a median duration of remission of 17.3 vs. 5.4 months and a median OS of 12.5 vs. 8.6 months [11]. The CRc among *FLT3*-WT patients was 67% vs. 26%, with median duration of response 18.4 vs. 13.4 months and median OS of 14.7 vs. 10.1 months [6]. In patients treated with VEN+AZA, CRc in AML patients with *FLT3-ITD* and *FLT3-TKD* mutations was 63% and 77% and median OS was 9.9 and 19.2 months [11]. The retrospective analysis of 127 ND AML patients treated with VEN-based regimens (mostly VEN+AZA) showed a similar rate of responses among *FLT3*-mutant and *FLT3*-WT patients (CRc rate of 79% vs. 61%, respectively; OS of 14.0 vs. 19.9 months, respectively) [12].

Patients with isolated *NPM1* mutations represent one of the groups of patients with the most favorable responses to VEN-based therapy. As mentioned above, the VEN+AZA combination improved CRc in *NPM1*-mutant AMLs from 24% to 67% [2]. Molecular measurable residual disease (MRD) by mutation-specific reverse transcription quantitative polymerase chain reaction (RT-qPCR) is strongly predictive of outcomes in patients with *NPM1*-mutant AMLs treated with IC [13]. Othman et al. explored the prognostic impact of molecular MRD in a cohort of 76 *NPM1*-mutant AML patients who achieved CR following treatment with VEN+HMA or VEN+low-dose cytarabine (VEN+LDAC) [14]. Of these patients, 58% achieved molecular MRD negativity and 18% achieved a reduction of ≥4 log_10_ from baseline, with no difference between the two groups of patients (VEN+HMA or VEN+LDAC) [8]. Patients achieving MRD negativity by the end of cycle 4 of treatment displayed a 2-year survival of 84% compared to 46% for patients with MRD positivity [14]. On multivariate analyses, molecular MRD negativity was the strongest prognostic factor [14]. Twenty-two patients who electively discontinued VEN-based therapy while in sustained MRD-negative remission achieved a 2-year treatment-free remission of 88% [14]. This last finding raises the problem of maintenance therapy in *NPM1*-mutant AML patients who achieved a durable complete molecular response. A subset of *NPM1-* and/or *IDH1*/*IDH2*-mutant AMLs receiving ≥12 months of VEN-based therapy can experience durable treatment-free remission after cessation of therapy [15]. A similar observation was made by Garciaz et al. showing that discontinuation VEN and/or AZA in AML patients (including *NPM1*-mutant) was associated with sustained responses and long-term survival rates [16]. In the absence of established criteria for selection of *NPM1*-mutant AML in remission for maintenance or for discontinuation of therapy and simple observation, it can be proposed according to Molica et al. [17] that: patients with isolated *NPM1* mutations sustained MRD negativity for >6–12 months, and stable recovery of hematological parameters may represent appropriate candidates for observation and potential treatment discontinuation; patients with comutations increasing relapse risk, such a *FLT3-ITD* with high allelic ration, *TP53* and *ASXL1* and with incomplete or unstable MRD responses are candidates for maintenance therapy.

*NRAS*/*KRAS* and *FLT3* mutations can affect the response of AMLs to VEN+HMAs. A recent study evaluated the impact of *NPM1*, *NRAS*/*KRAS* and *FLT3-ITD* mutations in a real-world cohort of 803 ND AML patients with a median age of 76 years treated with VEN+AZA; 80% patients were favorable and 20% intermediate-risk per ELN 2024 [18]. The presence of *NPM1*^mut^ was associated with significantly longer OS compared to *NPM1*^WT^; *RAS*^mut^ was associated with shorter OS compared to *RAS*^WT^, irrespective of *NRAS* or *KRAS*; the presence of *FLT3-ITD* did not affect outcome [18]. The stratification of *NPM1*^mut^ patients according to the presence of *RAS* or *FLT3-ITD* comutations showed that 2-year OS of *NPM1*^mut^/*RAS*^mut^ was inferior to that of *NPM1*^mut^/*RAS*^WT^ (27% vs. 51%, respectively), while the presence of *FLT3-ITD* comutations did not impact outcome [18].

Several studies have explored the efficacy of VEN-based regimens in *TP53*-mutant AML patients. Kim et al. evaluated the outcomes of *TP53*-mutant AMLs (31% of total) in a group of 121 AML patients (median age 72 years) receiving frontline treatment based on VEN+DEC [19]. *TP53*-mutant patients were more likely to have t-AML compared to those with *TP53*-WT (43% vs. 11%, respectively) and adverse ELN cytogenetic risk (92% vs. 27%, respectively) [19]. The CRc rate in the whole cohort of patients was 69% with MRD negativity (as assessed by flow cytometry) in 42% of cases. Patients with *TP53* mutations had a CRc rate of 54% with MRD negativity in 19% of cases, compared to a CRc rate of 76% with MRD negativity in 52% of cases in *TP53*-WT patients [19]. Sixty-day mortality was much higher among *TP53*-mutant than *TP53*-WT patients (26% vs. 4%); patients with *TP53* mutations had shorter OS than *TP53*-WT patients (5.2 vs. 19.4 months, respectively), as well as shorter EFS (3.4 vs. 18.9 months, respectively) [20].

DiNardo and coworkers explored the molecular patterns of response and treatment failure after frontline VEN+HMA or VEN+LDAC combinations and observed that *TP53* mutations are absent among patients displaying a durable remission but are enriched among patients unresponsive with primary resistance or those with acquired resistance after an initial response [21]. Pollyea and coworkers have analyzed the outcomes in patients with poor-risk cytogenetics with or without *TP53* mutations treated with VEN+AZA or AZA alone in the context of the VIALE-A clinical trial [22]. The results of this analysis showed that: VEN+AZA improved remission rates but not duration of response or OS compared with azacitidine alone; in low-risk cytogenetics and *TP53*-WT patients, VEN+AZA resulted in higher remission rates and longer OS and duration of response than AZA alone, with outcomes comparable to those observed in similarly treated patients with intermediate-risk cytogenetics [22]. Toxicities were comparable in *TP53*-WT and *TP53*-mutant patients [22].

Bader and coworkers have reported the retrospective analysis of a large set of *TP53*-mutant AML patients treated either with HMAs alone (50 patients) or VEN+HMAs (104 patients) [23]. These two groups of patients were comparable in age, cytogenetic profile and frequency of biallelic *TP53* alterations [23]. The median EFS and the median OS were not significantly different between the HMA and VEN+HMA groups (5.07 vs. 3.67 months, respectively, and 9.23 vs. 7.3 months, respectively); the median duration of response was higher in the VEN+HMA group compared to the HMA group (15.6 vs. 7.9 months, respectively) [23].

A retrospective study on the analysis of the outcomes of 91 *TP53*-mutant AMLs with a median age of 72 years treated with either IC or VEN+HMA and subdivided according to hit status (single-hit at diagnosis or double-hit) was carried out [24]. There was no significant difference in OS by treatment regimen (5.8 months in the IC group and 6.9 months in the VEN+HMA group) [24]. However, within the population that received IC, the patients with single-hit status had significantly longer mOS compared to double-hit (18.2 vs. 3.0 months, respectively), while no difference by hit status was observed in patients treated with VEN+HMA (7.4 months vs. 6.9 months) [24].

Other studies have evaluated in *TP53*-mutated and in high-risk AMLs a therapeutic approach based on a metronomic weekly dosing of a low dose of decitabine and VEN [25]. Mechanistically, metronomic dosing relies on terminal differentiation, rather than cytotoxicity, making it an attractive therapeutic regimen for *TP53*-mutant AML and other high-risk AMLs. Goldfinger explored the safety and the efficacy of this treatment in 21 high-risk AML patients with an age ≥75 years not candidate for any intensive chemotherapy treatment (five of these patients are *TP53*-mutant) [25]. Rockwell et al. reported the outcomes of 40 patients with *TP53*-mutated myeloid malignancies (14 AML and 26 MDS), 82% with biallelic *TP53* mutations and all low ELN risk [26]. Among AML patients, 70% achieved a CR and, among MDS patients, 49% achieved a CR [26]. For the entire cohort, OS was 11.3 months, 11.6 months for AML and 9.9 months for MDS patients [26]. Six patients underwent allo-HSCT, with an OS of 16 months [26]. The median OS observed in this study compares favorably with currently approved cytotoxic dosing of VEN+HMA [26].

A recent, single-arm retrospective study including 33 high-risk AML/MDS patients in CR with MRD negativity who underwent allo-HSCT and received VEN combined with a metronomic dose of DEC and interferon for prevention of post-transplant relapse found: 24-month OS and EFS were 92.4% and 83%, respectively; 24-month cumulative incidence of relapse and non-relapse mortality were 13.9% and 3.6%, respectively [27]. These observations support the utility of this treatment as an optimized maintenance strategy for relapse prevention post-allo-HSCT [27].

The mechanisms responsible for the poor response of *TP53^mut^* AMLs to VEN-based regimens were poorly explored. Vanttinen et al. have explored the mechanisms of resistance of *TP53^mut^* AMLs to VEN. Particularly, these authors explored a group of 16 AML patients, eight with *TP53^mut^* and eight with *TP53*^WT^ AMLs [28]. *TP53^mut^* blasts displayed a tendency to lower expression of BCL-2 compared to *TP53^WT^* AMLs [28]. Importantly, VEN+AZA-refractory AMLs exhibited significantly higher BCL-X_L_ expression compared to VEN+AZA-responsive *TP53^mut^* patients [28].

As discussed above, AMLs with *IDH* mutations represent one of the groups associated with the best responses to VEN+HMA therapy. Pollyea et al. performed a cumulative analysis of the *IDH1*/*IDH2*-mutant AML patients enrolled in the phase III VIALE-A and phase Ib M15-358 clinical studies [29]. The comparison of the outcomes of patients treated with VEN+AZA vs. those treated with AZA alone (VEN+AZA/AZA) showed that: among patients with *IDH1*/*IDH2* mutations CRc rate was 79%/11%, median duration of remission was 29.5/9.5 months, mOS was 24.5/6.2 months; among patients without *IDH1*/*IDH2* mutations CRc rate was 63%/31%, median duration of remission 17.5/10.3 months and mOS 12.3/10.1 months; among patients with *IDH1* mutations CRc was 66.7%/9.1% and mOS 15.2/2.2 months; among *IDH2* mutant patients, not reached (NR)/13.0 months [29]. In a second study, the same authors in a study including both ND and R/R AML patients showed that the combination of VEN+AZA resulted in high rates of responses, with durable remission and remarkable OS in both ND and R/R patients (42.2 vs. 15.8 months, respectively); 90% of responding patients were MRD-negative by flow cytometry, but the IDH mutations remained detectable in the majority of patients; MRD negativity by flow cytometry was associated with a clear survival benefit compared to patients who remained MRD-positive after therapy [30].

### 2.2. Responses in a Real-World Setting

The use of novel therapeutics in routine clinical practice may differ from that tested in the registration clinical trials that have led to their approval. Therefore, several clinical studies have evaluated the safety and the efficacy of VEN+HMA in a real-world setting.

The REVIVAL trial is a prospective clinical study aiming to demonstrate the effectiveness of VEN-containing therapy in the real world [31]. This study reported the outcomes of 209 ND IC-ineligible AML patients with a median age of 75 years; the large majority (94.7%) of these patients were treated with VEN+AZA, while only 2.9% of patients received VEN+DEC [31]. At a median follow-up of 22.5 months, mOS was 11.7 months and CRc rate was 65.2% [31]. When these patients were stratified according to VIALE-A original eligibility criteria, mOS was 17.8 months for patients meeting eligibility criteria and 10.7 months for patients who did not [31]. The results of this prospective observational study support the effectiveness of VEN-containing therapy in a real-world setting [31].

Ohtman and coworkers reported real-world outcomes of 654 patients treated in 53 UK hospitals with either VEN+AZA (587 patients) or LDAC (67 patients); the median age was 73 years [31]. Patients receiving VEN+AZA had a CRc rate of 67%, 30-day and 60-day mortality of 5% and 8%, respectively and a mOS of 13.6 months [32]. Mutations in *NPM1*, *RUNX1*, *STAG2* and *IDH2* were associated with improved survival, while age, s-AML and t-AML, trisomy 8, *MECOM* rearrangements, complex karyotype, *ASXL1* and *KIT* mutations were associated with poorer survival [32].

Brandwein and coworkers explored all AML patients (105) treated with VEN+AZA at a single institution in a real-world setting [33]. The ORR was 66% (highest for *NPM1* (78%), *IDH1*/*2* (82%) and lowest with *TP53* mutations (40%)), and the mOS was 9.6 months and 16.3 months for patients with CRc. On multivariate analysis *FLT3-ITD*/*RAS*/*TP53* mutations were associated with inferior OS [33]. The mOS of *FLT-ITD* patients was significantly lower than that observed for *FLT3-WT* patients (8.1 months vs. 16.0 months, respectively) [33].

Solana-Altabella and coworkers performed a systematic review and meta-analysis of unfit AML patients who have received VEN-based treatment, including 73 studies and more than 7000 treated patients [33]. The weighted mOS was 10.3 months, significantly lower than the mOS of 14.7 months observed in the VIALE-A trial; mean CRc rate was 58.2% and was comparable to the CRc rate of 66.4% observed in the VIALE-A trial; early death rates at 30 and 60 days were 5% and 13%, respectively; 15.4% of patients proceeded to allo-HSCT [34]. These observations suggest that an optimization of patient selection, dosing regimens, and supportive care is required to improve outcomes for VEN-treated patients in the context of real-world studies [34].

### 2.3. Genetic Risk Stratification in AML Patients Treated with Venetoclax and Hypomethylating Agents

The results of these studies have led to revising the risk classification system for AML patients undergoing treatment with VEN+AZA or other hypomethylating agents. In fact, the European Leukemia Network risk classification system was designed to predict outcomes of AML patients undergoing intensive chemotherapy treatment but was unable to offer an accurate prediction for AML patients undergoing treatment with VEN+HMAs [35]. To bypass this limitation, using clinical and biological data on patients treated with VEN+AZA in the context of the VIALE-A trial, Dohner and coworkers have proposed a four-gene prognostic gene signature (mPRS) predicting more accurately the outcomes of AML patients undergoing treatment with VEN+AZA [36]. This molecular prognostic risk signature uses *NRAS*/*KRAS*, *FLT3-ITD* and *TP53* mutations to stratify AML patients undergoing treatment with VEN+AZA [36]. This molecular prognostic risk signature uses *NRAS*/*KRAS*, *FLT3-ITD* and *TP53* mutations to stratify AML patients into three groups: lower benefit (mOS 5.5 months), characterized by the presence of *TP53* mutations; intermediate benefit mOS 12.1 months) characterized by the presence of *NRAS*/*KRAS* or *FLT3-ITD* mutations; higher benefit (mOS 26.5 months) characterized by the absence of the above-mentioned mutations [36].

The retrospective analysis of 159 AML patients who were treated with VEN+HMA showed that patients with an intermediate-benefit profile, enriched in *FLT3-ITD* and *NRAS*/*KRAS* mutations, display a mOS of 12 months, significantly shorter than the survival of 30 months observed for the higher-benefit group; patients in the lower-benefit group had lower incidence of *NPM1*, *ASXL1*, *RUNX1* and *IDH2* mutations [37].

Given the limitations of the ELN 2017 and ELN 2022 in predicting the risk of older AML patients treated with non-intensive chemotherapy treatments, a group of experts proposed a new risk classification (ELN 2024) for these patients [38]. According to this classification, the adverse-risk group includes *TP53*-mutated AMLs; the intermediate-risk group includes *FLT3-ITD*, and/or *KRAS*, and/or *NRAS*-mutated, *TP53*-WT; the favorable group includes different subtypes, including *NPM1*-mutant AMLs without *FLT3-ITD*, *NRAS*, *KRAS* and *TP53* mutations, *IDH2* mutation without *FLT3-ITD*, *NRAS*, *KRAS* and *TP53* mutations, *IDH1* mutation without *TP53* mutations, *DDX41* mutation and other molecular abnormalities without *FLT3-ITD*, *NRAS*, *KRAS* and *TP53* mutations [38].

Data from a real-world setting confirm the ability of the mPRS risk stratification system in terms of prediction of OS and EFS in the setting of newly diagnosed AML and also suggest its potential role for refractory/relapsed patients [39]. Interestingly, the analysis of minimal residual disease (MRD) showed that among patients achieving CRc only a part (46%) of those in the higher-benefit group achieved an MRD-negative condition [39]. Furthermore, a recent real-world study of UK patients treated with VEN+AZA showed that the new ELN 2024 performed better than the ELN 2022 [32].

The BEAT AML Program proposed a BEAT AML ELN-refined risk stratification for older adult AML patients undergoing lower-intensity therapy [40]. This classification model incorporates *IDH2*, *KRAS*, *MLL2* and *TP53* into the ELN 2022 classification to refine risk stratification among patients aged ≥60 years who received low-intensity treatments [40]. The BEAT AML 2024 classification performed better than the ELN 2022, particularly allowing a better stratification of the group of patients classified as adverse-risk in ELN 2022.

The evaluation of new older AML patients allowed incorporation of new additional criteria for the development of an improved ELN 2024 risk stratification classification (ELN 2024 refined) [41]. The ELN 2024 refined allowed the classification of three groups of patients: a favorable group (mutated *NPM1*, *IDH1*, *IDH2*, *DDX41* and wild-type *NRAS*, *KRAS*, *PTPN11*, *FLT3-ITD* and *TP53*), an intermediate group (mutated *FLT3-ITD*, *NRAS* and other mutations not classified and wild-type *KRAS*, *PTPN11* and *TP53*) and an adverse-risk group (mutated *KRAS*, *PTPN11* or *TP53*) [41]. Favorable-risk patients with mutations in *DDX41*, *NPM1* or *IDH1*/*2* display a particularly favorable survival (mOS 34.3 months), while patients with other mutations that are currently considered as favorable-risk but without mutations in *DDX41*, *NPM1* or *IDH1*/*2* have survival comparable to that observed for ELN 2024 intermediate-risk [41]. Compared to ELN 2024, ELN 2024 refined improved the classification of favorable- and intermediate-risk AMLs.

Hoff et al. comparatively evaluated the three risk stratification models mPRS, ELN 2024 refined and BEAT AML and reached the conclusion that all improved the risk classification of AML patients compared to ELN 2022 but all were equally unable to predict outcomes for all patients [42].

Gongat and coworkers developed a prognostic scoring system based on genetic signatures for response and survival of AML patients treated with VEN+HMAs, considering the mutational status of favorable mutations (*NPM1*, *IDH2* and *DDX41*) and unfavorable mutations (*FLT3-ITD*, *TP53* and *RUNX1*); in multivariate analysis, risk factors for inferior survival included failure to achieve CR, adverse karyotype, *TP53* mutations and the absence of *IDH2* mutations; this risk stratification model performed better than the ELN genetic risk model [43]. Based on these observations, Mayo genetic risk models were developed for ND AML patients treated with VEN+AZA, offering pre-treatment and response-based prognostic tools [44]. Particularly, for patients achieving a CRc response, adverse karyotype, *KRAS* mutations, *KMT2A* rearrangements and *IDH2*-WT predict inferior survival [44].

Recently, a new risk stratification model, called PRISM, was proposed to better predict overall survival in AML patients undergoing VEN-based treatments [45]. The PRISM model integrates multiple variables, including age, sex, s-AML, ELN 2022 complex karyotype and other adverse-risk cytogenetic abnormalities, adverse mutations (*KRAS*, *PTPN11*, *FLT3-ITD*, *JAK2*, *ASXL1* and *TP53*) and favorable mutations *(IDH2*, *RUNX1*, *CEBPA*, *BCOR*, *IDH1*, *SF3B1*) [45]. The PRISM model scores defined four risk groups: low (L), moderate (M), high (H) and very high (VH). The application of this model to a large cohort of AML patients who have received VEN-based treatment (VEN+HMA) showed that median OS by PRISM risk groups L/M/H/VH was 29.6, 17.6, 11.3 and 5.6 months [44]. PRISM risk groups improved OS versus mPRS [45].

A recent study carried out in 128 ND AML patients who received treatment with VEN+DEC in a real-world setting showed that, according to a multivariate analysis, neither ELN 2022 nor ELN 2024 significantly predicted RFS and OS [45]. However, a modified ELN 2024 risk model added complex karyotype (CK) to TP53 in the adverse-risk group [46]. This mELN 2024 risk model was associated with the best predictive ability for both EFS and OS [46].

### 2.4. The Sensitivity of Leukemic Blasts to VEN+HMAs Is Correlated with Cell Differentiation

Several studies have shown that not only the genotypic features of AML blasts but also their differentiation status influence sensitivity to VEN+HMA. An initial study on 100 AML patients characterized according to the morphologic FAB classification showed that AMLs with a more mature monocytic phenotype (classified as M5) are less sensitive to treatment with VEN+AZA; particularly, 62% of FAB M5 AMLs and 8% of non-FAB M5 AMLs are refractory to VEN+AZA [47]. According to these findings it was suggested that VEN+AZA response seems to correlate with differentiation stage, with phenotypically primitive AML blasts being more sensitive and monocytic differentiation more resistant to VEN+AZA [47].

Ex vivo drug assay confirmed that AMLs displaying a high proportion of monocytic cells (FAB M4/M5) are more resistant to VEN compared to those with phenotypically immature leukemic blasts (FAB M0/M1) [48]. Furthermore, AMLs with erythroid/megakaryocytic differentiation display resistance to VEN [49].

In spite of these observations, Waclawiczek and coworkers retrospectively analyzed 54 ND AML patients who received VEN+AZA; 26% of these patients did not achieve remission following VEN+AZA treatment and were considered clinically resistant [50]. Although cultured monocytic AML cells displayed upfront resistance, monocytic differentiation of AML blasts was not clinically predictive of resistance in this cohort of patients [50].

However, other studies supported the clinical relevance of AML monocytic differentiation as a phenotypic property associated with reduced clinical response to VEN+AZA treatment. Thus, Zhao and coworkers explored a group of 86 ND AML patients treated with VEN+AZA [51]. In these patients monocytic properties were defined using multiparametric flow cytometry; using this approach, monoblasts were defined as AML blasts with coexpression of ≥2 monocytic markers (CD14, CD36 and CD64) [49]. Patients with higher monoblast/CD45^+^ proportions had lower CRR and OS compared to those with lower monoblast/CD45^+^ frequencies [51]. Multivariable analysis supported monoblast^high^ status as an independent adverse prognostic factor for OS, with a particularly strong impact in ELN 2024 favorable-risk patients [51]. In fact, the presence of monoblast^high^ status identified a subgroup of AML patients classified as favorable-risk in ELN 2024 classification as a subgroup of patients with shorter OS [51].

Lachowiez et al. reported the analysis of two large cohorts of AML patients, including a total of 451 patients, treated with VEN+HMA; patients with monocytic AML had lower CRc rates (59% vs. 71%) and higher rates of 30-day mortality (11% vs. 5.5%) compared with patients with non-monocytic AML [50]. In the whole cohort of patients, monocytic differentiation was associated with shorter OS (7.8 months vs. 13.6 months) [52]. The negative impact of monocytic differentiation was also observed in subgroups of AMLs, characterized either by *NPM1* mutations or by *KRAS*, *PTPN11*, *FLT3-ITD* mutations [52]. Signaling pathway mutations (*NRAS*, *KRAS*, *PTPN11)* are enriched in patients with monocytic AML and have a clear prognostic implication for survival; these mutations can alter the level of antiapoptotic proteins, such as MCL1, promoting resistance to VEN+HMA and leukemic clone expansion [52].

The results of these studies highlight the importance of characterizing monocytic differentiation status and mutations in prognostic genes when choosing treatment for AML patients not eligible for IC.

Other studies have explored new strategies to improve the response of monocytic AMLs to VEN+HMA. The study of leukemia hierarchy composition in AML led to the identification of various developmental hierarchies, including also a hierarchy characterized by sensitivity to histone deacetylase inhibitors (HDACis) [53]. This observation provided a rationale for the clinical evaluation of the triplet drug based on VEN+AZA+Chidamide (a HDACi) (VAC regimen) in ND AML patients. Thus, Tan et al. evaluated 48 ND AML patients with mature monocytic phenotype, 28 receiving a VA regimen and 20 patients receiving a VAC regimen; at the end of the first cycle of treatment, ORR in VAC was 75% compared to 46.4% in VA; the MRD-negative status in patients achieving CR was 86.7% in the VAC group and 84% in the VA group; mOS was not reached in both groups of patients [54].

### 2.5. Ventoclax and Hypomethylating Agents in Relapsed/Refractory AML Patients

There is no standard salvage therapy in relapsed/refractory (R/R) AML patients, particularly in older patients. Feasible options involve various types of treatment, including VEN+HMAs. Response to VEN+HMA salvage treatment is reported in 20–40% of R/R AML patients, with a median OS ranging from 3.3 to 11.2 months [55].

Although the majority of R/R AML patients are unresponsive to VEN+HMA treatment, some molecular subgroups, including AMLs with *NPM1*, *IDH1* and *STAG2* mutations, are associated with high response rates and significant survival, while AML patients with high-risk molecular features such as adverse cytogenetics and mutations in *TP53*, *KRAS*/*NRAS*, *EZH2* and *SF3B1* are poorly responsive [56]. Other more recent studies have shown a particularly favorable response of R/R *NPM1*-mutant patients. Jimenez-Chillon reported 84% of responses in a group of 79 *NPM1*-mutant AML patients with *NPM1* molecular failure treated with VEN-based combinations [57]. Seventy-one percent of patients achieved MRD negativity after treatment [57]. Forty-one patients were bridged to allo-HSCT with no further therapy [57]. OS for the whole cohort at 2 years was 67% and EFS was 45% [57]. Presence of *FLT3-ITD* was associated with a lower response rate and shorter OS [57]. These observations have supported VEN-based therapies as a potentially effective treatment for molecular relapse in *NPM1*-mutated AML [57]. Another study carried out in 70 *NPM1*-mutant patients in molecular relapse after intensive chemotherapy showed a high rate of response, with a 2-year OS of 86% [58]. The majority of responding patients proceeded to allo-HSCT [58].

VEN-based salvage therapy may represent an effective and feasible bridge to transplant in AML R/R patients [59]. Propensity scoring matching comparing the results of this study on VEN+AZA-treated patients with a cohort of R/R AML patients treated with non-VEN-containing salvage therapy according to their treating physician’s choice (TPC), suggested a trend toward improved OS for VEN-treated patients: mOS of 15.8 months for VEN and 10.5 months for TPC; mEFS was significantly longer in the VEN cohort (8.0 months) than in the TPC cohort (3.7 months) (*p* = 0.06) [59]. Additionally, 73% of VEN-treated patients vs. 63% of TPC patients were bridged to allo-HSCT [59].

A risk stratification model to predict the outcomes of R/R AML patients undergoing treatment with low-intensity VEN-based regimens was proposed. Kruger et al., through the analysis of 163 R/R AML patients with a mean age of 70 years treated with VEN in combination either with HMA or LDAC, identified predictors of response and outcome. In these patients, ORR was 44%, CRc was 38%; mOS and mEFS were 8.4 months and 4.8 months, respectively [60]. Clinical predictors of survival included age, HMA pre-treatment, extramedullary disease manifestations and genomic profile. The mutational status of nine genes was associated with survival. AML patients were stratified into three risk groups based on the mutational profile of eight genes, generating three different categories, favorable (*STAG2*, *BCOR* or *SF3B1*-mutant), adverse (*TP53*, *FLT3*, *CBL*, *PTPN11* or *NF1*-mutant) and intermediate (none of the above) [60]. In a second study, the same authors evaluated 240 R/R AML patients who received treatment with VEN+HMA for the identification of prognostic risk factors at the genetic level; mutations in *NF1*, *PTPN11*, *FLT3* and *TP53* were identified as adverse-risk factors for inferior survival, whereas *SF3B1* mutations were identified as a favorable-risk factor [61]. These risk factors, including also extramedullary disease and prior HMA pre-treatment, were used to generate a risk model that allocated patients into three groups, favorable (mOS 21.4 months), intermediate (mOS 7.5 months) and adverse (mOS 4.5 months) [61]. This risk factor model was also predictive for newly diagnosed AML patients who received VEN+HMA treatment [61].

A multicenter, randomized controlled trial compared the efficacy of the mitoxantrone, etoposide, cytarabine (MEC) regimen versus the cladribine, VEN, low-dose cytarabine (CAV) regimen (in 55 R/R AML patients, 30 assigned to CAV regimen and 25 to MEC regimen) [62]. ORR was 53% in the CAV group and 32% in the MEC group; 1-year mOS was not reached in the CAV group and 67% in the MEC group; in the VEN-naïve patients, CAV achieved a significantly superior ORR of 66% vs. 32% with MEC [62].

Some studies have attempted to potentiate the response of R/R AML to VEN+HMA through the inclusion of an additional agent to this drug combination. Several studies focused on the addition of homoharringtonine (HOMO) to VEN+AZA. HOMO is an alkaloid extracted from the herb *Cephalotaxus mannii* found in southern China, inducing apoptosis of leukemic cells through reduction of MCL1 expression; in vitro and in vivo experimental studies showed a synergistic antileukemic effect of HOMO together with VEN. Thus, these observations have supported a clinical study based on the treatment regimen VAH, consisting of 14-day courses of VEN and 7-day courses of AZA and HOMO; VEN began at 100 mg on day 1 and increased stepwise over 3 days to reach the target dose of 400 mg per day from day 4 though day 14, AZA (75 mg/m^2^) and HOMO (1 mg/m^2^) were administered from day 1 to day 7 [63]. A retrospective analysis was performed on a group of 321 AML patients, 172 treated with VAH and 149 with VA treatment regimens. In comparison with VA, VAH enhanced the rates of CRc (44% vs. 66.3%, respectively), MRD negativity (34.8% vs. 59.3%, respectively), prolonged mOS (15.1 months vs. not reached) and EFS (3.8 months vs. 13.0 months) [61]. Furthermore, VAH mitigated the negative impact on VA efficacy of *FLT3-ITD* and *NRAS*/*KRAS* mutations [64]. A subanalysis of this study explored the response of AMLs with monocytic features (FAB M4 and M5) in comparison with non-monocytic AMLs (FAB M0, M1, M2 and M6) to VA and VAH [62]. In the cohort of patients treated with VS, the patients with monocytic-like AMLs displayed a lower response than those with non-monocytic features (ORR 47.4% vs. 65%; CRc 36.8% vs. 48.9%); however, in the cohort treated with VAH monocytic-like AMLs had similar response compared to those with non-monocytic features (ORR 74.4% vs. 73.3%; CRc 63.4% vs. 68.9%) [65].

However, no responses were observed by DiNardo and coworkers in a group of 22 heavily pre-treated AML patients with *RUNX1*-mutant AMLs, treated with a VAH regimen similar to that reported in the previous study [66].

Other studies evaluated the combination of pevonedistat (a small-molecule inhibitor of NEDD8) with VEN+AZA in the treatment of R/R AML patients showed an ORR of 46.7%; 80% of patients who achieved a CR were MRD-negative; four patients were bridged to allo-HSCT [67].

Several studies have evaluated VEN-based therapy in AML patients relapsing after allo-HSCT. A meta-analysis of studies carried out before 2025 included 337 AML patients relapsing after HSCT and treated with VEN+HMA (VEN+AZA 62% of cases; VEN+DEC 38% of cases) [68]. The 1-year OS was 29%; the pooled mortality was 29%; CRc rate was 39% [68].

Moukalled et al. reported a retrospective analysis on 224 AML patients relapsed after allo-HSCT present in the EBMT registry: 83 patients received VEN+AZA and 128 patients AZA alone [69]. The 1-year CR post-treatment was higher in the VEN+AZA group compared to the AZA group (44% vs. 26%), but the 2-year CR was similar (24% vs. 16%) [69]. In the group of patients with late relapse and without adverse cytogenetics, the rate of responses was better among patients treated with VEN+AZA compared to AZA alone [66]. It was estimated that around 20% of these patients treated with VEN+AZA may experience long-term survival [69].

Peters et al. reported the results observed in 26 AML patients relapsing after allo-HSCT and treated with VEN+AZA [70]. The ORR, CRc and CR rates were 69%, 57% and 38%, respectively; mEFS and mOS were 4.8 months and 15.0 months, respectively [70]. A part of these patients received donor lymphocyte infusions and those receiving these infusions before the start of VEN+AZA administration had a better mOS [70].

Chiusolo performed a retrospective observational analysis on AML patients relapsing after allo-HSCT and treated with VEN+HMA, involving nine Italian centers [71]. An ORR of 36%, with 33% of CR, was reported [71]. Cytogenetic and molecular features influenced response rate and survival: *TP53* mutations and complex karyotype correlated with absent response to VEN+HMA, while *NPM1* mutations were associated with a better response; patients displaying a CRc response had better survival, while patients with *TP53* mutations and/or complex karyotype had low OS [71].

### 2.6. Venetoclax Plus HMAs in Adult AML Patients Versus Induction Chemotherapy

Xie and coworkers have retrospectively evaluated the safety and the efficacy of VEN with DEC in 42 adult adverse-risk AML patients (median age 39 years); 93% of these patients achieved a CRc and 7% of patients did not achieve CRc after two cycles of therapy; 79% of patients with CRc achieved MRD negativity, as assessed by multiparametric flow cytometry (MFC) (<10^−3^) [72]. At 12 months, OS and EFS were 82% and 61%, respectively; 86% of patients received allo-HSCT [72]. Patients with *RUNX1* mutations were particularly sensitive to this treatment [72].

Cherry et al. reported the retrospective analysis of the outcomes of 143 ND AML patients who received VEN+AZA compared to 179 patients who received intensive chemotherapy (IC) [73]. The median OS was 884 days for IC and 483 days for VEN+AZA; however, when a propensity-matched cohort with equivalent baseline features was used to compare outcomes, OS was 705 days for IC and not reached for VEN+AZA [73]. Variables that favored response to VEN+AZA over IC were *RUNX1* mutations, older age and adverse risk; the presence of intermediate-risk AML favored IC over VEN+AZA [73].

Another study based on the use of VEN plus DEC versus intensive chemotherapy compared two groups of patients, each composed of 85 patients of comparable age (median age 72 years for VEN+DEC group and 73 years for IC group) [74]. Propensity scores were used to match patients to minimize bias. The VEN+DEC group was associated with higher CRc rate than the IC group (81% vs. 52%, respectively), lower rate of relapse (34% vs. 56%, respectively), longer OS (12 vs. 4.5 months) and lower 30-day mortality (1% vs. 24%) [74]. In conclusion, for older AML patients, particularly those at high risk of treatment-related mortality, VEN+DEC offers better outcomes than IC.

A retrospective analysis in AML patients aged ≥65 years treated with VEN-based regimens or with IC showed comparable OS in the two groups but a higher response rate and a more favorable safety profile in the VEN-treated group [75].

**Figure 2:**
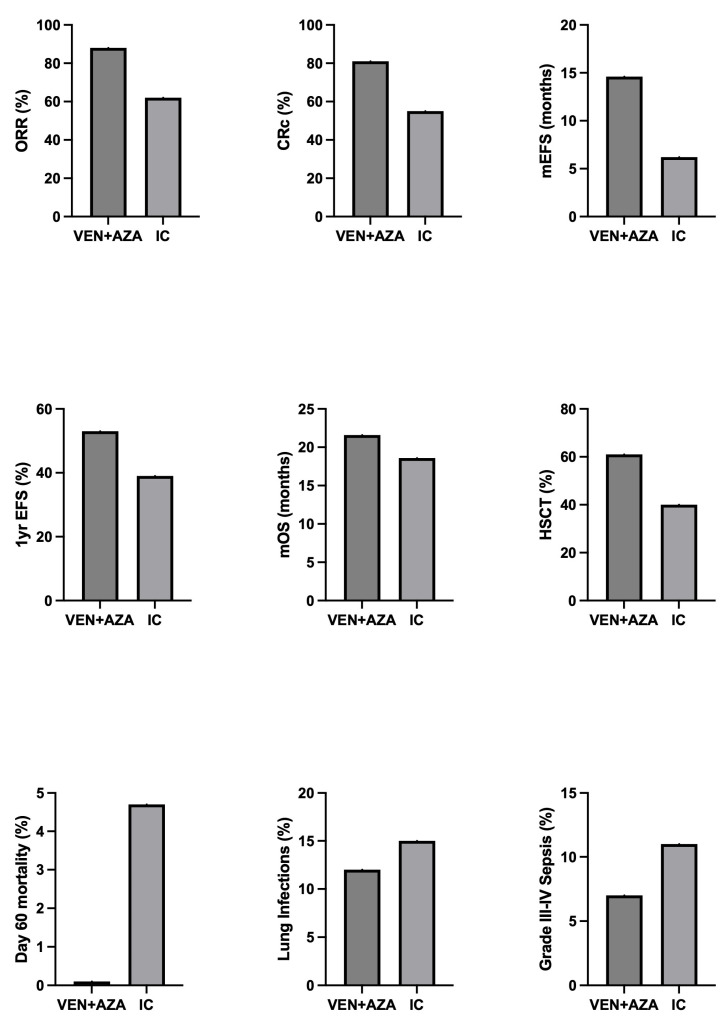
Outcomes observed in a phase II randomized clinical trial comparing the therapeutic activity of conventional Intensive Chemotherapy (IC, 7+3 regimen) to VEN+AZA among intensive-chemotherapy-eligible adult AML patients. Data reported in Faithi et al. [77].

Two randomized clinical trials compared VEN+AZA to conventional induction chemotherapy for newly diagnosed fit adults with AML. In a first study, Lu and coworkers enrolled 188 patients with ND AML with an age between 18 and 59 years who were eligible for IC and were randomized in a 1:1 ratio to receive either VEN+DEC or IC (idarubicin and cytarabine) [76]. CRc rate after induction therapy was 89% in the VEN+DEC arm and 79% in the IC arm; VEN-DEC displayed superior CRc in patients aged ≥40 years (91% vs. 75%) and those with adverse risk (91% vs. 42%) or epigenetic mutations (*IDH1*/*IDH2*, *DNMT3A*, *ASXL1* and *TET2* 91% vs. 67%) but lower CRc in *RUNX1-RUNXT1* fusion cases (44% vs. 88%) [76]. Patients in the VEN+DEC arm experienced fewer grade ≥3 infections and shorter severe thrombocytopenia duration than patients in the IC arm [76]. In conclusion, this study showed that VEN-DEC displayed non-inferior response rates with superior safety profile over IC in young patients with AML [76]. The second study was an open-label, multicenter phase II randomized clinical trial comparing the therapeutic activity of VEN+AZA to IC among IC-eligible patients aged ≥18 [77]. Patients with core binding fusions, *FLT3* mutations or *NPM1* mutations (unless aged ≥ 60) were excluded. Patients were allowed to proceed to transplant on both arms following response to treatment. One hundred and seventy-two patients were randomized to the two treatments in a 1:1 ratio and their median age was 64 for the VEN+AZA arm and 65 for the IC arm. The rate of CRc was higher in the VEN+AZA group than in the IC group (81% vs. 55%); procession to allo-HSCT was higher for the VEN+AZA group than for the IC arm (61% vs. 40%); EFS was higher in the VEN+AZA arm than in the IC arm (53% vs. 39%); MEFS was 14.6 months in the VEN+AZA arm compared to 6.2 months in the IC arm [77]. mOS showed comparable levels in the VEN+AZA group (21.5 months) compared to the IC group (18.6 months); however, the interpretation of mOS values is made complex by the frequent cross-over events (67% shift from IC to VEN+AZA and 47% from VEN+AZA to IC) in refractory or relapsing patients [77] (Figure 2). AZA+VEN treatment led to numerically fewer serious infectious complications [77].

Fang et al. reported the results of a randomized phase II trial comparing in elderly AML patients, aged 60–75 years, three different therapeutic regimens: standard IC (arm A), VEN+AZA (arm B) and VEN+Chemotherapy (arm C) [78]. A total of 102 AML patients were enrolled. The following results were observed in arms A, B and C, respectively: CR rates of 37.5, 47.2, and 61.8%; CRc rates of 40.6%, 60.9% and 61.8%; mortality rates within 30 days of 12.5%, 2.8% and 11.7%; EFS of 7.1, 5.7 and 4.5 months; mOS of 10.4, not reached and 14.1 months. Importantly, within the ELN 2022 adverse-risk subgroup EFS and OS were improved in patients treated with VEN+AZA compared to those receiving IC [78].

Wan and coworkers have retrospectively analyzed the outcomes of 188 AML patients with an age between 18 and 64 years and with myelodysplasia-related changes who received treatment with VEN+AZA or 7+3 IC [70]. Patients were classified into an MR-G group (only MR mutations, including *SRFSF2*, *SF3B1*, *U2AF1*, *ZRSR2*, *EZH2*, *BCOR*, *STAG2* and *RUNX1* mutations), MR-C group (only MR cytogenetic abnormalities) and MR-G/C group (both MR gene mutations and cytogenetic abnormalities) [79]. After induction therapy, both the ORR and CRc rates of the VEN+HMA cohort were significantly higher than those of the IC cohort (75% vs. 46% for ORR and 61% and 32% for CRc) [79]. Furthermore, MRD-negative CR rate was higher in the VEN+HMA group than in the IC group (46% vs. 19%, respectively) [79]. The EFS was longer in the VEN+HMA group than in the IC group (not reached vs. 1.2 months, respectively) [79]. Of patients, 56% in the VEN+HMA cohort and 59% in the IC cohort received allo-HSCT [79].

Other studies have compared the response to VEN+HMA treatment and IC in molecular subgroups of AML patients. A multicenter retrospective cohort study of ND 221 *NPM1*-mutant AML patients evaluated the outcomes following treatment with IC (147 patients) or VEN+AZA (74 patients) [80]. CRc was similar for IC and VEN+HMA (55% vs. 74%, respectively); in unselected patients 24-month OS was 59% with IC and 38% with VEN+HMA and in patients aged 60–75 years, 24-month OS was 60% with IC and 44% with VEN+HMA [80]. In subgroup analysis, patients with normal cytogenetics and without *FLT3-ITD* might benefit from IC compared to VEN+HMA [80]. In multivariate analysis, OS was not statistically different between IC and VEN+HMA [80].

Dunn and coworkers retrospectively analyzed the outcomes observed in 141 *NPM1*-mutant AML patients (median age, 51 years) who received treatment with IC or VEN+AZA (patients not eligible for IC). In the IC and VEN+AZA groups the CRc rate (97% vs. 95%, respectively) and 2-year OS (88% vs. 71%, respectively) were similar [81].

Another study confirmed the findings of these studies, showing that outcomes of patients aged 60 to 75 years with *NPM1*-mutated AMLs are similar between IC and VEN+HMA [82]. Furthermore, patients who underwent allo-HSCT had excellent outcomes, regardless of whether their firstline treatment regimen was based on IC or VEN+HMA [82].

VINCENT, a randomized-controlled clinical trial will evaluate VEN+AZA versus IC in patients aged 18–70 years with ND, *NPM1*-mutated AML [83].

Other studies have compared the outcomes of AML patients who received CPX-351 as induction treatment compared to those treated with VEN+HMA. A retrospective meta-analysis was carried out on 1852 older ND AML patients treated with CPX-351 (a liposomal, dual-drug formulation of cytarabine and daunorubicin in a 5:1 molar ratio) or VEN+HMA as frontline therapy. The mean age of patients was 63.5 years in the CPX-351 group and 73 years in the VEN+HMA group [84]. mOS and CRc rate were similar in the two groups of patients; 30-day and 60-day mortality were also similar in these patients [84]. A similar conclusion was reached by Fathima and coworkers through the retrospective analysis of 600 ND AML patients treated either with CPX-351 (19%) or VEN+HMA (81) [85]. The outcomes of these patients were similar in CPX-351- and VEN+HMA-treated patients: ORR 55% vs. 60%; mOS 10 vs. 13 months; CRc rate 45% vs. 60% [85]. An analysis of subsets of AML patients showed: similar rates of response in AMLs with adverse karyotype, *NPM1^mut^*, *TP53^mut^*, *IDH1*^mut^, *IDH2^mut^*; mOS in patients with post-MDS AML was higher in the VEN+HMA group than in the CPX-351 group (12 vs. 7 months); mOS in patients with *SF3B1* mutations was lower in the VEN+HMA group compared to the CPX-351 group (14 months vs. not reached) [85].

Several recent studies have explored whether VEN+HMA treatment may improve the outcome of various subsets of high-risk AML patients compared to IC. Boussi et al. performed a retrospective analysis of a large cohort of AML patients with deletion of chromosome 7 (-7) and del 5 or 5q (-5/del5q), comparing the outcomes between IC- and VEN+HMA-treated patients [86]. mEFS and mOS were similar in the two groups of patients, treated with VEN+HMA or IC [86]. OS improved only in patients who received allo-HSCT, irrespective of frontline therapy [86]. Aguirre et al. have recently reported the retrospective analysis of 358 ND AML patients with very-high-risk cytogenetics (vHRC), defined by complex karyotype (CK), monosomal karyotype (MK) and inv3/t(3;3), who received either IC (40%) or VEN+HMA as frontline therapy [87]. *TP53* mutations occurred in 51% of these patients. mOS for these patients was 8 months and was similar in the two groups of patients treated with IC or VEN+HMA [87]. On multivariate analysis, older age, inv(3)/t(3;3) and *TP53^mut^* independently predicted OS [87]. Given the equivalent efficacy and similar early mortality, VEN+HMA represents a valuable frontline option for patients with high-risk AML, including patients intended for allo-HSCT.

Bouligny et al. retrospectively analyzed 50 older AML patients with *IDH* mutations: 48% received IC, 36% VEN+HMA and 16% HMA monotherapy [88]. The mOS in the IC and VEN+HMA cohorts was comparable (14.1 months vs. 12.2 months, respectively) and was significantly higher than that observed in the group treated with HMA monotherapy (6 months) [88]. Bewersdorf et al. have retrospectively analyzed the outcomes of 151 *IDH*-mutant AML patients >60 years who received a treatment based on IC (81 patients) or VERN+AZA (70 patients) [89]. CRc and 24-month OS were similar for patients treated with IC or VEN+AZA, including subgroups of patients, such as age 60–75 years, type of *IDH* mutation, ELN risk category and allo-HSCT receipt [89]. Multivariate analysis showed that treatment type did not impact OS [89]. These findings provide evidence supporting a comparable efficacy of VEN+HMA and IC in older adults with *IDH^mut^* AML.

Real-world studies based on a comparison of VEN+HMAs and intensive chemotherapy have provided variable results. Thus, some studies have evidenced a better OS and more favorable safety profile in patients treated with VEN+HMAs [90,91], while other studies have shown a better OS in patients treated with IC [92,93]; furthermore, other studies indicate similar outcomes in patients treated with VEN+HMA and IC [88]. These discrepancies may be related to the heterogeneity of patients and to the difficulty of comparing homogeneous groups of patients for baseline characteristics. Interestingly, Chen and coworkers have performed a genotype-guided comparison of VEN+HMA versus IC in a real-world context, showing that patients aged ≥60 years or harboring *FLT3-ITD*, *DNMT3A* and *TET2* mutations exhibit superior responses to VEN+HMA [91].

### 2.7. Venetoclax and Low-Dose Citarabine (VEN+LDAC)

Venetoclax and low-dose cytarabine (VEN+LDAC) represent an effective and well-tolerated treatment option for older AML patients who are ineligible for intensive chemotherapy. Response rates to LDAC as frontline therapy in older AML patients are poor and thus there is a strong rationale to potentiate the effects of LDAC though its association with VEN. Thus, a phase II clinical trial of VEN combined with LDAC in AML patients showed a CRc rate of 54%, with median OS of about 10 months, that compare favorably with historical response rates and survival observed in studies of LDAC monotherapy in AML patients [94]. A phase III randomized trial (VIALE-C) compared the outcomes in 215 elderly AML patients randomized in a 2:1 ratio to receive VEN+LDAC or Placebo+LDAC [88]. The CRc rate of 48% observed in VEN+LDAC was markedly higher than the CRc rate of 13% observed in patients treated with LDAC monotherapy; the mOS for patients on VEN+LDAC was 8.4 months compared to 4.1 months for LDAC alone [95]. Longer-term follow-up confirmed that patients receiving VEN+LDAC had longer OS than those receiving Placebo+LDAC; CRc repsonses in the VEN+LDAC arm were durable, with 11% remaining in remission for >2 years; for patients with *NPM1* mutations treated with VEN+LDAC, OS at 24 months was about 50%; outcomes of patients with *TP53* mutations were poor [96].

A meta-analysis including five studies and a total of 493 patients confirmed that patients receiving VEN+LDAC have a significantly higher OS and CR compared to LDAC alone [97].

A recent study provided criteria for risk stratification of AML patients who received VEN+LDAC treatment [91]. Analyzing a cohort of 139 patients treated with VEN+LDAC, Wei et al. reported that neither the ELN 2022 nor the mPRS classifier models adequately stratified prognosis of these patients [98]. The reason for the failure of the mPRS model to predict prognosis of VEN+LDAC-treated patients is that in these patients *FLT3-ITD* and *NRAS*/*KRAS* mutations are equally distributed in the intermediate- and in the high-benefit groups [91]. Particularly, in these patients *NPM1*-mutant AMLs display a significantly better OS than *NPM1-WT* AMLs (mOS 29.6 vs. 11.5 months) and the presence of *FLT3-ITD* or *RAS* mutations does not affect outcomes [98]. AMLs with *TP53* mutations and AMLs with complex karyotype alterations, either with or without concomitant *TP53* mutations, are poorly responsive and have low mOS [98].

Other studies have evaluated a triplet combination involving VEN+AZA+LDAC (defined as VAA). A first study evaluated this drug combination in 12 AML (four with R/R disease and five with s-AML) patients with a median age of 65 years. The ORR was 91.7%, including 83.5% CR and 8.3% CRi; mOS and mPFS were 15.3 months and 10.8 months, respectively [99].

Han and coworkers reported the results of a randomized controlled clinical trial involving the enrollment of 78 adult AML patients (age >60 years) randomized 1:1 to receive treatment with VEN+AZA (VA) or VEN+AZA+LDAC (VAA) [100]. The estimated mOS for the VAA group was 17.6 months compared to 14.6 months in the VA group; in the VAA group, 61.5% of patients achieved CR with MRD negativity and 20.5% CR with MRD positivity, while in the VA group only 37.5% of patients achieved CR with MRD negativity; eight patients in the VAA group and one patient in the VA group underwent allo-HSCT [93]. Interestingly, AMLs with monocytic features, including FAB M4 and M5, reached a CR more frequently in the VAA group than in the VA group (69% vs. 28%, respectively). In another recent study VEN+AZA was combined with low-dose cytarabine and idarubicin (VAIA regimen) and retrospectively compared with standard cytarabine and idarubicin (IA) induction [101]. Particularly, 26 patients treated with VAIA were compared to 52 patients treated with IA, with matched clinical and molecular features [101]. The VAIA cohort displayed a significantly higher CR rates and MRD negativity compared to the IA cohort (84% vs. 61% for CR; 81% vs. 54% for MRD negativity); furthermore, 1-year OS and 1-year LFS were improved in the VAIA cohort compared to the IA cohort (77% vs. 58% for OS and 65% vs. 46% for LFS) [101].

### 2.8. Venetoclax in Association with Intensive Chemotherapy

VEN in association with intensive chemotherapy (such as 7+3 or FLAG-IDA or CLIA) has shown promising results in treating AML, particularly in high-risk or R/R cases.

The 7+3 regimen is the standard induction chemotherapy for ND AML combining 7 days of continuous intravenous cytarabine (Ara-C) with 3 days of an anthracycline (daunorubicin or idarubucin). Wang et al. evaluated the safety and the activity of VEN+7+3 cytarabine and daunorubicin chemotherapy (DAV regimen) in adults with AML in the context of a phase II, single-arm, trial; 33 patients aged 18–60 years with ND AML were enrolled [102]. The CRc rate was 97%, with 97% of patients reaching CR with MRD negativity in 97% of cases [102]. Grade 3 adverse events mostly involved hematological parameters, with 55% being cases of febrile neutropenia, 24% pneumonia and 12% sepsis [102]. No treatment-related deaths occurred [102]. In a second study, the same authors performed a propensity-score-matched analysis comparing the outcomes of the patients treated with DAV to a historical group treated with DA [103]. The group displayed a higher rate of CR than the DA group (90% vs. 55%, respectively) and a higher rate of MRD-negative CRs (96% vs. 62%, respectively) [103]. DAV treatment improved OS and EFS but not DFS [103].

A more recent study confirmed the findings of this study [104]. Thus, Mantzaris et al. have performed a phase Ib study exploring the combination of VEN (investigated with three different schedules of administration of either 7, 11 or 14 days) and 7+3 IC (Arac and daunorubicin) [104]. A total of 34 patients were enrolled with a CRc rate of 85.3% with 86.2% MRD negativity among patients who achieved CR [104]. With a median follow-up of 9.6 months, EFS, OS and duration of regimen were reached [104]. The rate of response was similar for all three different schedules of VEN administration [104].

Liu et al. reported a retrospective analysis on 63 ND AML patients with *FLT3-ITD* mutations who received DAV treatment [105]. The ORR was 97.7% with CR rate of 91.8%; 92.5% of patients achieved CR with undetectable MRD [105]. The 1-year OS, EFS and RFS were 73%, 59% and 59%, respectively [105]. Nineteen percent of patients proceeded to allo-HSCT [98]. Adverse events were similar to those reported in the study of Wang et al. [103].

Chua and coworkers in the context of the CAVEAT phase Ib study enrolled 85 patients aged ≥65 years with ND AML considered suitable for IC and treated with a modified 7+3 regimen limiting the administration of cytarabine to 5 days and idarubicin to 2 days; this 5+2 chemotherapy regimen was associated with VEN evaluated at various doses [106]. The CAVEAT induction regimen was well tolerated, with 4% mortality [99]. The ORR was 88%, with a mOS of 33.1 months [106]. Almost one third of patients did not relapse with a treatment-free remission of 17.9 months [106].

A phase Ib/II clinical study evaluated the safety and the efficacy of fludarabine, cytarabine, granulocyte colony stimulating factor, and idarubicin (FLAG-IDA) combined with VEN in ND and R/R AML patients. The phase Ib trial enrolled patients into two arms to evaluate response and time-to-event in ND AML (phase IIa) and R/R AML (phase IIb) [100]. The ORR for PIB, PIIA and PIIB were 75%, 97% and 70%, respectively, with 75%, 90% and 61%, respectively, achieving CRc; MRD negativity in patients who achieved a CR was 96% in ND AML and 69% in R/R AML [107]. Additionally, 69% of ND AML and 46% of R/R AML proceeded to allo-HSCT [107]. Recently, a long-term analysis of 138 patients (77 ND and 61 R/R) enrolled in this study showed that: in the ND cohort, the ORR was 97%, CRc rate 95%, MRD negativity by flow cytometry 90%, the 3-year OS and EFS rates 66% and 64%, respectively; in the R/R cohort, the ORR was 67%, CRc rate 41%, MRD negativity 74% and 3-year OS 51% [108]. Additionally, 71% of ND AML patients proceeded to allo-HSCT, compared to 51% of R/R patients [108]. Importantly, in ND AML patients, the rates of response to FLAG+IDA+VEN were similar in the three groups of risk-stratified patients according to ELN 2022: in the favorable, intermediate and adverse ELN groups CRc rates and MRD negativity were 100%, 91%, 97% and 92%, 90%, 88%, respectively [109].

Al-Shaibani et al. et al. retrospectively evaluated 44 AML patients (17 ND high-risk AMLs and 21 R/R AMLs) treated with FLAG-IDA+VEN [110]. Among ND AML patients, ORR was 71% with 75% achieving MRD negativity; OS and RFS were 9 months and 8 months, respectively; six patients proceeded to allo-HSCT [110]. Among R/R AML patients, ORR was 67%, with 67% achieving MRD negativity; mOS and RFS were 26 months and 15 months, respectively; 12 patients proceeded to allo-HSCT [110]. Ten patients experienced disease relapse after achieving remission with FLAG+IDA+VEN treatment [110].

A retrospective analysis on 53 R/R AML patients receiving salvage therapy with FLAG-IDA (35 patients) or FLAG-IDA+VEN (18 patients) showed a better EFS in patients treated with FLAG-IDA+VEN compared to those treated with FLAG-IDA [111].

Shahswar et al. in a retrospective study have compared safety and efficacy of FLAG-IDA without or with 7-day VEN in patients with R/R AML [112]. Thirty-seven patients received one course of FLAG-IDA+VEN and 81 patients FLAG-IDA. The ORR was significantly higher in FLAG-IDA+VEN compared to FLAG-IDA (78% vs. 47%), while MRD negativity was comparable at a similar proportion in responding patients (50% vs. 57%, respectively); 81% and 79% of patients proceeded to allo-HSCT after FLAG-IDA+VEN and FLAG-IDA, respectively [112]. EFS and OS were similar in the two groups of patients [112].

Raj et al. reported a retrospective analysis on 12 adolescents/young adults who received FLAG-IDA+VEN (11 with ND AML and one with t-AML); the median age of these patients was 20 years (from 2 to 25 years) [113]. All 12 patients experienced CR, 9/12 with MRD negativity; seven patients with CR proceeded to allo-HSCT; the 6-month EFS was 80.2% and 6-month OS rate was 88.8% [113]. These preliminary observations support prospective studies evaluating FLAG-IDA+VEN in pediatric/young AML patients [113].

A real-world study carried out by Federov et al. on 32 ND and R/R AML patients treated with FLAG-IDA with 7-day or 14-day VEN showed high response rates in both frontline and R/R settings; remission rates were high even in high-risk AML subtypes including adverse-risk ELN [114]. Response rates were comparable in patients treated with 7-day VEN and 14-day VEN [114]. Analysis of the safety profile supports a close monitoring of patients and favors the 7-day VEN regimen over the 14-day VEN regimen [114].

Bashey and coworkers comparatively evaluated 200 consecutive ND AML patients who underwent treatment with FLAG-IDA (154 patients) or FLAG+IDA+VEN (46 patients) [108]. The two groups of patients matched well in terms of their clinical characteristics, with 52% and 51% of patients pertaining to the NCCN adverse-risk classification among FLAG-IDA and FLAG-IDA+VEN-treated patients [115]. CR and CRc rates following a single cycle of treatment of FLAG-IDA or FLAG-IDA+VEN were 93% and 83% and 98% and 88%, respectively [115]. In NCCN low/intermediate-risk AML, CR rates and CRc rates were 91% vs. 75% and 97% vs. 83% [115]. For intermediate/low-risk patients 55% of patients induced with FLAG-IDA+VEN and 59% of those treated with FLAG-IDA proceeded to allo-HSCT [115]. The estimated 1-year LFS in the two groups of patients was similar (75% in FLAG-IDA+VEN group and 79% in FLAG-IDA group) [115]. These observations show that FLAG-IDA+VEN treatment resulted in a higher rate of CRc compared to FLAG-IDA in the low/intermediate-risk AML patients; the evaluation of the effects on OS requires a longer follow-up [115].

Schonrock and coworkers have developed a reduced-intensity regimen of FLAG-IDA and was evaluated without or with VEN in R/R AMLs showing a better safety profile and an efficacy comparable to that observed for a standard FLAG-IDA regimen [116].

A multicenter phase I/II study, GIMEMA 1718, investigated the safety and efficacy of VEN combined with fludarabine, cytarabine, idarubicin (V-FLAI regimen) as induction therapy for patients with non-low-risk AML aged <65 years and at intermediate or high ELN risk [117]. In the phase I trial the patients were randomly allocated to VEN 400 mg or VEN 600 mg cohorts [117]. A preliminary analysis of 57 patients showed that: safety and effectiveness were similar for VEN 400 mg and VEN 600 mg cohorts; CRc was achieved in 84% of patients; 60-day mortality rate was 58%; with a median follow-up of 20.6 months, 1-year OS, DFS and cumulative incidence of relapse were 71%, 66.2% and 24%, respectively [117]. Fifty-five more patients will be enrolled in part 2 of the study and will receive 400 mg VEN [117].

Recently, Ruhnke and coworkers reported the results of the phase I/II RELAX study evaluating high-dose cytarabine and mitoxantrone (HAM) in combination with VEN as a salvage therapy for 55 medically fit patients with R/R AML [118]. Forty-five percent of the enrolled patients had adverse-risk AML and 35% had previous allo-HASCT [118]. The CRc rate was 75%. The most common adverse events were sepsis and pneumonia; potential treatment-related deaths were reported in four patients [118]. The 24-month OS was 56% and 24-month EFS was 58%; among patients who achieved a CRc condition, 24% were MRD-negative by flow cytometry and 41% by RT-PCR; 88% of patients achieving CRc proceeded to allo-HSCT [118]. Importantly, compared with historical salvage treatment with HAM alone, HAM+VEN improved outcomes, allowing a high rate of patients to proceed to allo-HSCT.

Other studies have explored the intensive chemotherapy regimen cladiribine, high-dose cytarabine and idarubicin (CLIA) in association with VEN [119]. A phase II clinical trial evaluated in 50 ND AML patients (median age 48 years) the CLIA+VEN regimen with an ORR of 94%, with 82% of MRD negativity; at a median follow-up of 13.5 months, the median duration of response, EFS and OS were not reached; at 12 months, DOR was 74%, EFS 68% and OS 85% [119]. The most common adverse events were related to hematological complications; two patients died of infectious complications [119].

A single-institution study confirmed the efficacy of the CLIA+VEN regimen in 15 ND AML patients with 1-year PFS and OS both of 93.3% [120]. Lee and coworkers in a retrospective analysis compared the outcomes observed in 20 ND AML patients who received CLIA+VEN to a historical cohort of AML patients who received 7+3 induction chemotherapy [121]. CLIA+VEN was associated with a higher rate of CRc (90% vs. 54.8%) and MRD negativity (93.8% vs. 60.9%) compared to the 7+3 regimen [121]. Furthermore, OS was significantly improved in the CLIA+VEN cohort compared to the 7+3 cohort [121].

CPX-351, a nanoscale liposome that includes a fixed 5:1 molar ratio of cytarabine and daunorubicin, is approved as frontline therapy in patients with ND t-AML and AML with myelodysplasia-related changes (AML-MRC). A phase II clinical study reported the results observed in 17 ND AML patients (59% with AML-MRC, 47% with CK and 29% with *TP53^mut^*) treated with CPX-351+VEN [122]. ORR was 82%, with a CRc rate of 71%; in AML-MRC patients, ORR was 100%, with CRc of 90%; patients with prior HMA exposure had a CRc rate of 65%; in all patients, mOS was 12.8 months and in those with AML-MRC mOS was 17.9 months [122]. Eighty-six percent of responding patients proceeded to allo-HSCT [122].

Croden and coworkers reported the outcomes of *IDH*-mutant AML patients treated either with FLAG-IDA+VEN or CLIA+VEN [123]. In this retrospective analysis, a total of 124 AML patients were treated with frontline VEN+IC: 99 were *IDH*-WT, 10 *IDH1*^mut^ and 15 *IDH2*^mut^. The ORR was 96% in *IDH*-WT, 80% in *IDH1*^mut^ and 100% in *IDH2*^mut^; 38% of *IDH1*^mut^ and 27% of *IDH2*^mut^ AML patients proceeded to allo-HSCT; OS at 3 years for *IDH*-WT, *IDH1*^mut^ and *IDH2*^mut^ AMLs was 76%, 60% and 61%, respectively [123].

In conclusion, these studies support IC+VEN as an effective regimen in both ND and R/R AML patients. Particularly, the FLAG-IDA+VEN regimen appears to be risk agnostic, with similar effects in ELN favorable-, intermediate- and adverse-risk groups. However, future randomized studies will be required to assess both in ND and R/R AML patients whether the association of VEN with IC regimens improves OS compared to IC alone and to define AML subtypes which may benefit from this treatment.

### 2.9. Venetoclax-Based Treatments as Maintenance Therapy in AML

VEN-based regimens have been investigated as maintenance therapy post-chemotherapy and post-HSCT. The rationale of a maintenance therapy in AML patients who have reached a remission condition is that the majority of patients relapse and will die from leukemia if not bridged to allo-HSCT.

The main clinical objective of maintenance therapy consists in administering low, well-tolerated doses to sustain remission and eventually to eliminate the small fraction of residual disease.

In this context, some studies have supported the efficacy of HMAs and VEN as maintenance therapy. The phase III clinical trial QUAZAR AML-001 evaluated oral AZA as a treatment suitable to prolong OS in patients in patients >55 years with AML in first CR/CRi after IC who were not candidates for allo-HSCT [123]. After a median follow-up of 41.2 months, AZA was shown to improve OS over placebo from 14.8 to 24.7 months, as well as EFS from 4.8 to 10.2 months [124]. Molecular subgroup analyses showed a pronounced benefit for *NPM1*-mutant AML patients with an improvement of OS from 15.9 months in the placebo group to 47.2 months in the AZA group [125]. Long-term follow-up analysis showed that the survival curves of the AZA and placebo arms remained separate in favor of AZA up to 80 months, but after this time the curves appeared to narrow [126]. Oral AZA was approved as maintenance therapy in patients who achieve CR after IC but are unable to complete intensive consolidation therapy.

However, a phase III randomized study of AZA maintenance vs. observation in high-risk AML and MDS patients failed to show any significant improvement of RFS and OS by AZA treatment [127].

Several studies have explored the safety and the efficacy of VEN+HMA post-chemotherapy or post-allo-HSCT. In a phase II clinical trial on 35 AML patients in remission after IC or low-intensity induction chemotherapy, Bazinet and coworkers explored the role of low-dose AZA in association with VEN as maintenance therapy. After a median follow-up of 23.3 months, the median RFS was not reached in the whole cohort and 30 months in the cohort of patients who received prior low-intensity therapy [128]. Two-year RFS was 65% in the whole cohort: 71% in the patients who received prior IC and 52% in those who received low-intensity chemotherapy [128]. Eight patients proceeded to allo-HSCT. Patients with *NPM1* and *IDH1*/*IDH2* mutations had favorable outcomes, while those considered adverse-risk by ELN relapsed during maintenance therapy [128]. Although the comparison with the QUAZAR AML-001 trial is difficult for some remarkable differences in the baseline characteristics of the patients enrolled in these two trials, the RFS rate observed in this trial compares favorably to the RFS observed in the QUAZAR AML-001 trial [128].

Recently, Medawar and coworkers retrospectively reported the outcomes of 29 patients with ND-AML who received VEN+HMA maintenance therapy after IC and CR/CRi; median age at diagnosis was 62 years; 58% of patients had ELN 2022 adverse risk [128]. Most common reasons for switching to VEN+HMA were post-induction MRD positivity and decline in performance post-induction therapy [129]. mOS was 37.7 months for the whole cohort, NR for patients who proceeded to allo-HSCT and 19.3 months for patients not proceeding to HSCT [129]. RFS was not reached for the whole cohort and for patients who proceeded to HSCT and 11.2 months for patients not proceeding to HSCT [129]. Of the five MDR^+^ patients, all converted to MRD negativity after one cycle of maintenance therapy [129].

VEN-based treatments were also evaluated in high-risk AML patients who underwent allo-HSCT as maintenance therapy aiming to prevent or to delay the risk of relapse post-transplant. Kent evaluated the safety and the efficacy of VEN administered to 49 AML patients at high risk of relapse; at 1-year post-HSCT, OS and RFS were 70% and 67%, respectively [130]. VEN administration was well tolerated and 88% of patients completed the full year of planned therapy [130]. Oran et al. reported the results of a phase II trial evaluating the safety and the efficacy of VEN+AZA as maintenance therapy post allo-HSCT in 58 high-risk AML patients [131]. At 1 year and 2 years, RFS was 63.3% and 54.1%, respectively; at 1 year and 2 years, OS was 82.5% and 56%, respectively [131]. Despite frequent hematological toxicity, the treatment was usually well tolerated, with two cases of non-relapse mortality [131].

Garcia et al. have conducted a phase I trial assessing safety and efficacy of prophylactic maintenance with VEN+AZA after reduced-intensity conditioning allo-HSCT for high-risk MDS and AML patients [132]. This maintenance treatment was safe, with low rates of infections and chronic GVHD and minimal impact on T-cell immune reconstitution [131]. The 2-year OS, PFS, non-relapse mortality and cumulative incidence of relapse rates were 67%, 59%, 0% and 41%, respectively [132].

Diebold et al. compared outcomes of adult patients with AML and MDS undergoing reduced-intensity conditioning and receiving post-HSCT low-dose VEN+HMA maintenance to a matched cohort of RIC allo-HSCT recipients that did not receive maintenance therapy [133]. The median RFS and OS for the maintenance group vs. no maintenance group were not reached vs. 21.9 months and not reached vs. 31.3 months, respectively [133].

In conclusion, VEN+HMA is a safe and effective regimen for maintenance therapy after IC or allo-HSCT; however, randomized clinical trials are required to assess its superiority with respect to oral AZA, an approved maintenance treatment.

### 2.10. Treatment of FLT3-Mutant AMLs with Combinations of Venetoclax and FLT3 Inhibitors

As above discussed, older AML patients with *FLT3-ITD* mutations treated with VEN+AZA do not have a significant advantage with a median OS around 10 months [11]. The combination of gilteritinib (GIL), an FLT3i, with AZA failed to improve mOS compared to AZA alone (9.8 months vs. 8.8 months, respectively) [134].

Pre-clinical studies showed that VEN combines synergistically with FLT3 inhibitors to effectively target *FLT3-ITD*^+^ AML cells, resulting in a strong antitumor activity and providing mechanistic rationale for clinical studies [135]. Interestingly, high-throughput drug screening identified GIL and VEN as a highly synergistic drug combination also acting in *FLT3-WT* AML [136]. The GIL-VEN combination inhibited the expression of the MCL-1 antiapoptotic protein and decreased the viability of VEN-AZA-resistant cells in vitro and in vivo [135].

A phase Ib clinical study evaluated the combination of VEN+GIL in R/R *FLT3*^+^ AML, reporting a high rate of *FLT3* molecular response (60%) in patients who achieved CRc; mOS was 10 months [137]. The treatment resulted in consistent myelosuppression, requiring dose interruptions to mitigate cytopenias [137].

According to these initial studies it was hypothesized that triplet therapy containing an FLT3i, VEN+HMA or FLT3i+VEN+LDAC may potentiate the antileukemic activity, further improving outcomes [136]. In an initial clinical study, a small cohort of older/unfit patients with ND *FLT3*^+^ AML were treated with a triplet drug combination including an FLT3i, VEN and DEC; of the 12 patients who received the triplet drug combination, 92% of patients achieved CRc, with MRD negativity in 91% of patients who achieved CRc [138]. In a second study, Yilmaz and coworkers retrospectively analyzed 87 *FLT3*^mut^ patients who received either a doublet treatment based on low-intensity chemotherapy (LDAC or LDAC-cladribine) in association with an FLT3i (60 patients) or a triplet treatment based on LIC, FLT3i and VEN (27 patients) [139]. Triplet therapy was associated with a greater rate of CR (67% vs. 32%), CRc (95% vs. 70%), and FLT3 negativity (96% vs. 54%) than doublets [138]. With a median follow-up of 24 months, mOS was longer in triplet than doublet regimens (not reached versus 9.5 months) [139].

These initial studies have strongly supported the development of triplet regimens for the treatment of older/unfit *FLT3^mut^* AML patients [140].

A phase I/II study evaluated AZA/GIL and VEN in two cohorts of AML patients: patients with ND *FLT3^mut^* AML who are unfit for IC and R/R AML patients [141]. With the frontline therapy, the CRc rate was 96% and 60% of patients achieved MRD positivity at <5 × 10^−5^; with a median follow-up of 19.3 months, the 18-month RFS and OS were 71% and 72%, respectively [141]. In the R/R cohort, CRc rate was 27% [141].

Recently, a long-term follow-up of ND AML patients enrolled in this study was reported. With a median follow-up of 41.5 months, the mRFS and MOS were 23.4 months and 29.7 months, with a 3-year RFS and OS of 63% and 46%, respectively [142]. MRD negativity was achieved in 93% of patients [142]. Among patients with *FLT3-ITD* mutations, mRFS and mOS were 17 and 21.8 months, while in those with *FLT3-TKD* mutations they were 38.5 months and not reached [142]. Among 11 patients who relapsed, eight had *FLT3-ITD* and three had *FLT3-TKD* mutations at baseline; at relapse, six of nine patients no longer had detectable *FLT3* mutations [142].

The ongoing phase I/II VICEROY study is a multicenter, randomized, phase I/II study evaluating the VEN-AZA-GIL triplet using two different VEN dosages, 200 or 400 mg VEN [143]. CRc rate was 90% at VEN 200 and 91% at VEN 400; 12-month OS was 64% at VEN 200 and 77% at VEN 400 [143]. Based on the safety response profile, VEN 400 was the recommended phase II dose [143].

Arora et al. have recently reported the initial results of a phase II clinical trial involving the treatment of adult AML patients with adverse-risk *FLT3-WT* AML [144]. The analysis of the first 15 patients showed: CRc rates in *TP53*-mut and *TP53*-WT of 44% and 67%, respectively; in seven responders, MRD negativity by flow cytometry was 71%; four patients underwent allo-HSCT; with a follow-up of 7.6 months, no patients in CRc relapsed [144].

In a retrospective cohort study, Jiang et al. have evaluated the safety and the efficacy of the triplet regimen VEN, AZA, GIL in 48 *FLT3-ITD*-mutated AML patients with R/R disease or persistent MRD, as a bridge to allo-HSCT [145]. After one cycle of therapy, 81.3% of patients achieved CRc, with 70% of MRD negativity; no TRM was observed; 1-year OS and RFS were 83.3% and 75%, respectively; 1-year relapse and non-relapse mortality were 14.6% and 10.4%, respectively [145].

**Figure 3:**
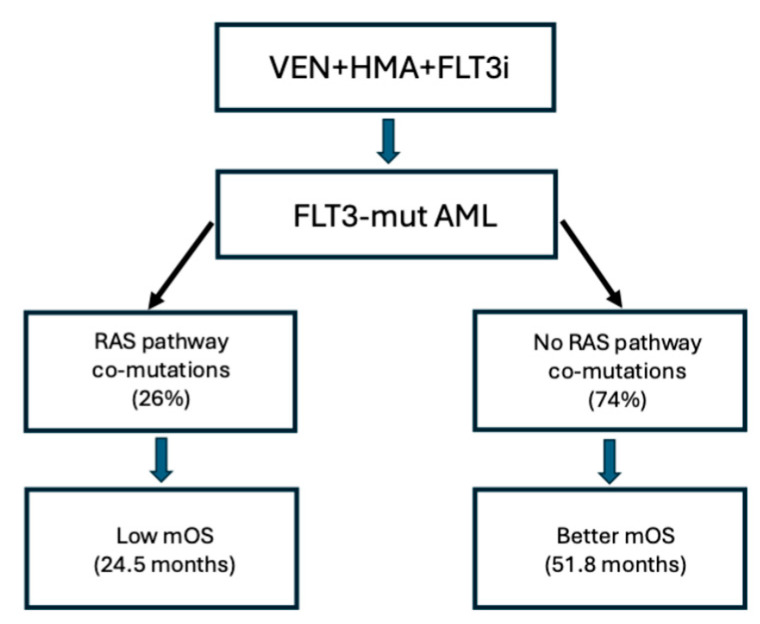
Median OS in *FLT3*-mutant AML patients without or with baseline *RAS* pathway mutations who received a triplet regimen based on VEN+HMA+FLT3i. Patients without baseline *RAS* pathway mutations have a significantly better mOS (51.8 months) compared to those with *RAS* pathway mutations (24.5 months), following triplet regimen treatment. Data are reported in Short et al. [146].

Short and coworkers reported a retrospective analysis on 73 ND AML patients who received a frontline FLT3-inhibitor-containing triplet regimen [146]. The CRc rate was 93%, with 60% MRD negativity (48% in *FLT3-ITD^+^* AML) after two cycles and 90% (70% in *FLT3-ITD*^+^) after four cycles; the 3-year RFS and OS for *FLT3-ITD* AMLs were 38% and 45% and for *FLT3-TKD* AMLs 76% and 76%, respectively [146]. Baseline *RAS* mutations were associated with poor long-term survival: mOS of 24.5 months in AMLs with *RAS* pathway mutations compared to a mOS of 51.8 months in AMLs without *RAS* mutations [146] (Figure 3).

**Figure 4:**
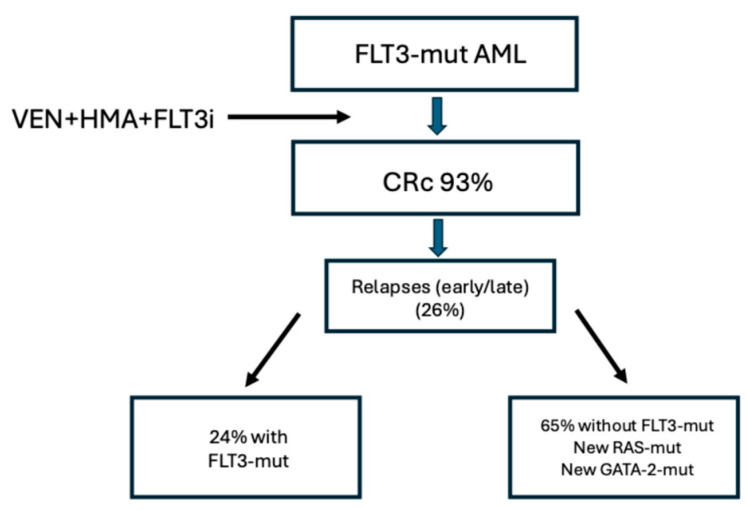
New *RAS* and *GATA-2* mutations in *FLT3*-mutant patients who relapsed with *FLT3*-WT or *FLT3-mut* AML after frontline treatment with triplet VEN+HMA+FLT3i. 73 patients with newly diagnosed FLT3-mutated AML received a frontline FLT3-inhibitor-containing triplet regimen and 93% of them achieved a CRc; 26% of these patients relapsed at early or late times: 24% of these relapsing patients relapsed with *FLT3 mutations* and 65% with leukemic cells without *FLT3* mutations but acquiring new *RAS* and *GATA-2* mutations. Data reported in Short et al. [146].

*FLT3-WT* relapses were observed in 65% of relapsing patients without *FLT3* mutations and new *RAS* mutations were detected in 24% of relapses and new *GATA-2* mutations in 18% of relapses, both exclusively in patients with *FLT3-WT* relapses [145] (Figure 4).

In an analysis of the molecular profile of 80 AML patients who relapsed after front-line therapy with IC+FLT3i, HMA+VEN+FLT3i or low-intensity therapy+FLT3i, Arera et al. showed that: relapses with *FLT3*-mutant loss were more frequent in patients who received IC+FLT3i or triplet therapy than in those who received LIT+FLT3i; at relapse, patients who received HMA+VEN+FLT3i had higher rates of emergent *RAS* pathway and DNA methylation mutations compared to those who received IC+FLT3i or LIT+FLT3i [147]. Relapses after allo-HSCT had lower rates of emergent non-*FLT3* mutations [147].

The combination of VEN, DEC and quizartinib (QUIZ) was evaluated in a phase II clinical study specific to patients with *FLT3-ITD* mutation, 30 ND and 46 R/R AMLs [147]. Among ND AML patients, 94% achieved a CRc and 75% of responders were MRD-negative by flow cytometry and 61% by RT-PCR; 30-day mortality was 3%; 30% of patients underwent allo-HSCT; with a median follow-up of 17 months, mOS was not reached [148]. Among R/R AML patients, 61% achieved CRc and 30% of responders were MRD-negative by flow cytometry and 33% by RT-PCR; mOS was 6.1 months and 1-year OS was 20% [149].

A triplet regimen using an LDAC+VEN backbone in combination or not with midostaurin was evaluated in a randomized phase II trial of patients with AML with intermediate-risk cytogenetics; patients were randomized to receive LDAC+VEN+MID or LDAC+VEN [149]. MID was administered sequentially to LDAC to reduce the risk of myelosuppression. The trial enrolled a total of 120 patients in a 2:1 randomization between LDAC+VEN+MID vs. LDAC+VEN; 22% of patients were *FLT3-ITD*-positive in the LDAC+VEN+MID group [150]. In the subgroup of *FLT3-ITD*-positive patients, CRc was observed in 82% of patients in the LDAC+VEN+MID group, compared to 57% in the LDAC+VEN group; *FLT3-ITD* MRD clearance was observed in 60% of patients in the LDAC+VEN+MID arm and 50% in the LDAC+VEN arm; mOS was 16.8 months, with 1-year OS of 57% [150].

### 2.11. Treatment of IDH-Mutant AML with Venetoclax-Based Regimens and IDH Inhibitors

*IDH*-mutant AMLs represent a heterogeneous group of AMLs whose incidence increases with age and whose prognosis is dependent on the presence of frequent comutations, mainly represented by *NPM1*, *DNMT3A* and *FLT3-ITD*.

The mutant *IDH1* inhibitors ivosidenib (IVO) and olutasidenib (OLU) and mutant *IDH2* inhibitor enasidenib (ENA) promote the differentiation of *IDH*-mutant AMLs and are approved for the treatment of *IDH1* and *IDH2*-mutant AMLs. Furthermore, IVO in combination with AZA (IVO+AZA) is approved for patients with ND *IDH1^mut^* AMLs who are ineligible for IC, based on the results of the phase III AGILE study showing a mOS of 29.3 months in the group treated with IVO+AZA compared to 7.9 months in the group treated with AZA+Placebo [151].

Pre-clinical studies have shown a synergism between VEN and IDH inhibitors, thus providing the rationale for IDH-based drug doublets (VEN+IDHi) or triplets (VEN+HMA+IDHi).

The pooled analysis of two prospective phase II trials of VEN+AZA+IVO for ND *IDH1^mut^* AML and DEC-cedazuridine (DEC-C)+VEN+IVO or +ENA was recently reported [152]. The two studies enrolled a total of 60 IC-ineligible patients. The CRc rate was 92%, with an ORR of 95%; 87% of responding patients achieved MRD negativity by flow cytometry after two cycles of treatment [142]. With a median follow-up of 27.4 months, mOS was not reached. The 2-year OS was 69% with a 2-year cumulative incidence of relapse of 24% [152]. Molecular analysis of patients who relapsed after IDH triplet therapy showed that 50% of *IDH2^mut^* and 83% of *IDH1^mut^* AML relapses were IDH-WT, thus suggesting that triplet therapy was able to eradicate the *IDH^mut^* clone and that most relapses are due to evolution or progression of a separate leukemic clone [152]. Although the safety profile of this triplet regimen was favorable, dose adjustments and supportive care were required in many patients, particularly after six cycles of treatments [152]. Given the good outcomes observed in this study, a phase III randomized trial (EVOLVE-1) comparing the triplet to a doublet regimen is now enrolling.

The phase Ib/II AML-150 clinical enrolled 105 AML *IDH^mut^* patients (62 ND, 43 R/R) undergoing treatment with a triplet regimen based on DEC-C, VEN and ENA or IVO [143]. Median age of patients was 71 years. The CRc rate was 87% in the ND cohort, with 91% MRD negativity in responders; among ND patients, CRc rate was 91% in treatment-naïve patients and 73% in those with therapy or secondary AMLs. For patients with R/R disease, CRc rate was 60% with MRD negativity of 58%. For the ND patients mOS was not reached, with a 2-year OS of 63% and 79% if patients with therapy or secondary AML are excluded [144]. For R/R patients, mOS was 1.3 months and 2-year OS was 34% [153]. Twenty percent of patients proceeded to allo-HSCT. Early mortality was low, with two deaths within 30 days.

Longitudinal genomic analyses of *IDH*-mutant AML patients undergoing treatment with IDH inhibitors in various combinations have contributed to understanding the molecular mechanisms underlying resistance to this treatment. The analysis of resistance mechanisms in 60 AML patients who received treatment with IDH1 and IDH2 inhibitors showed that leukemia stemness is a major driver of primary resistance to IDH inhibitors, while selection of mutations in *RUNX1*/*CEBPA* or *RAS*/*RTK* pathway genes is the main driver of acquired resistance to IDH inhibitors [154].

Lachowiez et al. reported the longitudinal molecular analysis of 31 patients with *IDH1*-mutant myeloid malignancies (14 ND AML, eight R/R AML and nine MDS/MPN) treated with either VEN+IVO (12 patients) or VEN+IVO+AZA (19 patients) [146]. CRc rate and 24-month OS with VEN+IVO+AZA vs. VEN+IVO were 90% vs. 83% and 75% vs. 58%, respectively; in ND AML patients 12-month OS was 90% vs. 50% with VEN+IVO+AZA or VEN+IVO, respectively [146]. *IDH1* mutation clearance in patients receiving >5 cycles of therapy was 86% in the VEN+IVO+AZA group and 43% in the VEN+IVO group [155]. Patients with methylation gene mutations (*DNMT3A*, *TET2*, *IDH2*) other than *IDH1* displayed improved survival compared to those with WT status [155]. Patients with mutations in signaling pathway genes (*FLT3*, *NRAS*, *KRAS*, *NF1*, *PTPN11*, *JAK*/*STAT*, *KIT*, *CSF3R*) showed a trend to shorter survival, particularly evident for those with RAS/RTK pathway signaling [155]. Patients with signaling pathway mutations appeared to benefit from treatment with VEN+IVO+AZA compared to VEN+IVO [155]. Longitudinal single-cell DNA sequencing studies showed the elimination of large leukemic clones containing *IDH1*/*NPM1*/*NRAS* mutations with VEN+IVO+AZA; at relapse, outgrowth of minor *IDH1+NPM1+NRAS+KRAS* mutation-containing clones was observed [155]. In other patients, there was elimination of *TP53*/*SF3B1* and *TP53* clones without co-occurring *IDH1* mutations [155]. These observations suggest that clonal composition is a major determinant of the response to VEN+IVO+AZA treatment [155]. At variance with patients treated with IDH inhibitor monotherapy, in patients treated with VEN+IVO combinations, emergent mutations in transcription factor genes rather than *RAS*/*RTK* signaling pathways were common at relapse [155].

Sirenko and coworkers have explored the clonal architecture of *IDH*-mutant AMLs at baseline and after treatment with IDH inhibitors alone or in association with chemotherapy or HMAs. Gene expression studies showed that *IDH*-mutant AMLs are characterized by expanded progenitor-like populations and an extended inflammatory phenotype: in samples with *NPM1* comutations are associated with a GMP phenotype, while *SRSF2* mutations with a MEP phenotype [156]. Each *IDH*-mutant AML was characterized by a consistent level of clonal heterogeneity; typical examples are given by the presence of an ancestral *IDH1* and *DNMT3A*-mutated clone and an *NRAS*-mutated subclone or an ancestral clone with *IDH2* and *DNMT3A* mutations and a subclone with *SRFS2* mutations [156]. Initiating mutations in epigenetic genes (*IDH* and *DNMT3A*) confer a stem-like, non-proliferative and inflammatory phenotype, while later mutations in signaling or splicing genes (*NRAS* and *SRFSF2*) are associated with proliferation, lineage skewing and impaired differentiation [156]. In responding patients, parental clones defined by *IDH* and *DNMT3A* mutations or copy number alterations respond to therapy by differentiation and reconstitution of hematopoiesis [156]. Relapse after therapy with IDH inhibitors is associated with selection of pre-existing minute subclones [156].

**Figure 5:**
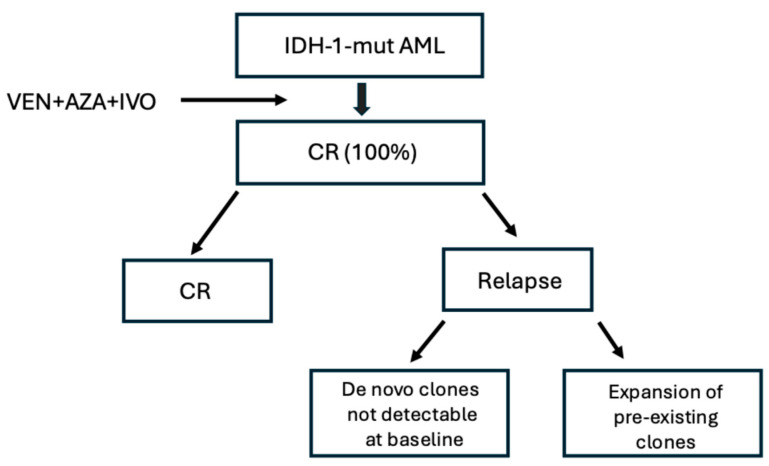
Clonal evolution of *IDH1*-mutant AMLs undergoing treatment with VEN+AZA+IVO. After an initial response observed in virtually all *IDH1*-mutant patients some patients remain in remission, while other patients relapse with an AML generated either by de novo clones not detectable at baseline or by expansion of minor clones existing at diagnosis.

Turkilji and coworkers used high-sensitivity single-cell genotyping and RNA-sequencing techniques for the characterization of the clonal evolution and transcriptional changes in bone marrow cells in eight *IDH1*-mutant AML patients who received treatment with VEN+AZA+IVO [157]. All the patients achieved a CR after triplet therapy; however, late outcomes of these patients were different, with two patients remaining in CR and six patients who relapsed 4 to 50 months from the initial therapy [157]. The clonal analysis of the mutational profile of the six patients who relapsed provided evidence that three patients developed novel driver mutations (*FLT3-ITD* or *SF3B1* or *RUNX1* mutations), while the remaining three patients developed outgrowth of pre-existing small clones with a stem cell phenotype and impaired differentiation, expanding after therapy [157] (Figure 5). These results indicate that the multiclonality of *IDH*-mutant AMLs represents a major determinant of the response to current treatments including IDH inhibitors and strategies must be developed to target these subclones responsible for disease progression.

### 2.12. Targeting CD123 with Anti-CD123 Agents in Combination with Venetoclax

CD123 (the alpha chain subunit of the inteleukin-3 receptor) is highly expressed in approximately 80% of AML cases, particularly on leukemic stem cells, making it a key therapeutic target [158,159]. CD123 overexpression on leukemic blasts is associated with increased proliferative activity, resistance to apoptosis and poor prognosis following chemotherapy induction treatment [160]. CD123 overexpression in AML is frequently associated with *FLT3-ITD* mutations [161,162]. CD123 is consistently expressed in *NPM1^mut^* AMLs and its overexpression is particularly evident in double-mutant *NPM1*/*FLTR3-ITD* AMLs. In these AMLs, CD123 expression if clearly higher on CD34^+^/CD38^−^ cells than on bulk leukemic cells [163].

CD123 is overexpressed on the surface of AML blasts compared to healthy HSCs, allowing for a therapeutic targeting; thus, various antibodies targeting CD123 have been developed and their potential use in the treatment of AML is under evaluation [159]. Pre-clinical studies have shown that VEN synergizes with antibodies targeting CD123 by potentiating apoptosis of leukemic cells [164]. On the other hand, a retrospective analysis on AML patients treated with VEN+HMA showed a significant antileukemic effect in AMLs overexpressing CD123 [165].

Pivekimab sunirine (PVEK) is a first-in-class antibody–drug conjugate comprising a high-affinity anti-CD123 antibody, a cleavable linker, and an indolinobenzodiazepine pseudodimer payload. In patients with CD123-positive AMLs, PVEK combined with VEN+AZA showed robust response rates with a manageable safety profile [166]. Using this triplet drug regimen, Daver et al. recently reported the outcomes observed in 49 ND CD123^+^ AML patients, with a mean age of 77 years, 51% with adverse risk and 35% with *TP53* mutations [166]. With a median follow-up of 10 months, 79% of patients achieved a CRc, with 90% MRD negativity among patients achieving CRc; 16% of patients were bridged to allo-HSCT [167]. Response rates were consistent across various mutational profiles tested [167]. This triplet regimen also showed consistent efficacy in a group of patients with blastic plasmocytoid dendritic cell neoplasms (BPCDNs), a rare and aggressive hematologic malignancy [168].

Another triplet drug regimen, based on tagroxofusp (composed of recombinant IL3 coupled to diphtheria toxin payload), AZA and VEN showed consistent efficacy in BPCDN patients, including R/R patients, with a high rate of patients bridged to allo-HSCT [169].

Finally, a recent study showed significant efficacy of a triplet regimen based on mipletimig, a CD123 × CD3 bispecific antibody, AZA and VEN in nine AML patients, including four patients with *TP53*^mut^; 78% of patients achieved a CR, all associated with MRD negativity [170].

## 3. Conclusions

The treatment of ND-AML patients considered unfit to receive IC has evolved, moving to treatments less toxic than chemotherapy, such as VEN and a HMA that is the current standard-of-care for these patients. VEN+HMA represents a basic backbone with flexible options regarding VEN dose and duration of cycles of treatment.

Recent studies also support the extension of this treatment to the therapy of adult AML patients fit for IC. However, additional randomization studies on a larger number of patients, comparing the VEN+HMA regimen to IC regimen, are needed. Furthermore, additional studies are required to determine the molecular subtypes that more clearly have survival benefits compared to standard IC. In fact, studies carried out in recent years have shown the high heterogeneity in treatment response and survival outcomes of patients with ND AM receiving VEN+HMA, associated with their profile of genetic alterations.

Other studies suggest the possible benefit deriving from various IC regimens combined with VEN, particularly in high-risk AML patients. As frontline therapy, IC plus VEN regimens may improve the number of high-risk patients who can be bridged to allo-HSCT. In R/R AML patients, IC+VEN regimens may improve OS and the number of patients bridged to allo-HSCT. However, randomized clinical studies, supported by concomitant genome analyses, are required to assess the superiority of IC+VEN with respect to IC in some subsets of AML patients.

Some studies have suggested the possible effectiveness of VEN-based regimens as maintenance therapy in AML patients who have undergone HSCT. Although several studies showed encouraging results, randomized clinical studies are required to demonstrate a better benefit from VEN-based regimens compared to AZA or to other approved drugs for maintenance therapy.

A last area of considerable interest is represented by the investigation of the combination of VEN+HMA with targeted therapies (FLT3, IDH1, IDH2 and Menin inhibitors) in patients with actionable mutations. The studies so far carried out have supported consistent efficacy with tolerable toxicity (often requiring dosing adjustments) of these triplet drug regimens. Ongoing randomized clinical trials are required to confirm the capacity of these triplet regimens to generate a therapeutic benefit in terms of improvements of response rates and overall survival for these various genomic subgroups of AML. Concomitantly, the development of new therapeutic strategies will be necessary to limit disease relapse and progression observed in a significant proportion of patients treated with triplet regimens through targeting of resistant subclones.

Current strategies involving VEN face significant limitations primarily revolving around safety and primary and secondary resistance. The safety concerns raised by VEN are mainly related to the high rate of hematological toxicity: the 28-day standard cycle of VEN administration is difficult due to frequent neutropenia and thrombocytopenia; thus, many patients cannot tolerate the full 28-day cycle and must receive only 14 or 7 days of treatment. Resistance mechanisms represent another important limitation of VEN-based regimens. Thus, despite their consistent efficacy, 20–30% of AML patients show primary resistance. Furthermore, despite high initial response rates, many AML patients develop resistance, leading to relapse. For refractory or relapsed patients there are very possible effective options. Finally, VEN causes rapid leukemic cell death, which can lead to tumor lysis syndrome, a potentially life-threating metabolic complication.

The future of studies on VEN will first consist in the achievement of randomized clinical trials to assess the benefit deriving from VEN’s combination with intensive chemotherapy in newly diagnosed and R/R patients and the inclusion of VEN in triplet regimens, in the post-transplant maintenance setting and in combination with targeted agents in some AML subsets. Furthermore, future therapeutic strategies have to attempt to overcome the limitations discussed above. Particularly, it will be of crucial importance to develop adequate strategies to overcome resistance mechanisms to VEN and evaluate the combination of VEN with other antiapoptotic inhibitors, such as MCL-1 inhibitors.

## Data Availability

No new data are created.
